# Layered Porous Nanocubes: Harnessing Trimetallic PBA@WS_2_–Phosphorus Hybrid Architecture for Efficient Oxygen Evolution

**DOI:** 10.1021/acsami.6c01187

**Published:** 2026-02-18

**Authors:** Poulami Mukherjee, Krishnamoorthy Sathiyan, Ronen Bar-Ziv, Koichi Higashimine, Toshiaki Taniike, Arie Borenstein, Tomer Zidki

**Affiliations:** † Department of Chemical Sciences and the Centers for Radical Reactions and Materials Research, 42732 Ariel University, Ariel 4077625, Israel; ‡ Graduate School of Advanced Science and Technology, Japan Advanced Institute of Science and Technology, 1-1 Asahidai, Nomi, Ishikawa 923-1292, Japan; § Institute of Advanced Energy, 34807 Kyoto University, Kyoto 611-0011, Japan; ∥ Department of Chemistry, Nuclear Research Centre, Negev, Beer-Sheva 84190, Israel; ⊥ The Center for Nano Materials and Technology, Japan Advanced Institute of Science and Technology, 1-1 Asahidai, Nomi, Ishikawa 923-1292, Japan

**Keywords:** oxygen evolution reaction, Prussian blue analogs, WS_2_ nanosheets, phosphidation, core−shell, M−S–P heterojunction

## Abstract

The rational assembly of multiple components in electrocatalyst design offers a promising strategy to enhance sluggish oxygen evolution reaction (OER) kinetics. However, the maximum utilization of such complex systems requires an understanding of each component’s role and precise nanoscale control to uncover meaningful structure–activity relationships. This work presents a stepwise approach to designing a high-performance OER precatalyst by combining trimetallic Prussian blue analogs (PBAs), WS_2_ nanosheets, and phosphorus doping, thereby forming a cooperative network within the designed M–S–P heterojunction. Each step addresses a specific challenge, ranging from structural templating and active-site enrichment to electronic modulation and mass-transport optimization. The heterojunction establishes a unique interfacial architecture in which both S and P anions are electronically bridged to transition-metal centers. Multiple metals in a core–shell configuration introduce redox diversity, enabling cooperative electron transfer and distributing the oxidative burden across neighboring sites. The dual anions play distinct yet complementary roles: the P anion accelerates proton transfer, while the S anion improves adsorption kinetics by donating electrons to stabilize OH* and OOH* intermediates. Under operating conditions, the precatalyst undergoes in situ surface reconstruction, forming the oxidized active catalyst that delivers an overpotential of 280 mV at 10 mA cm^–2^, a Tafel slope of 70 mV dec^–1^, and an impressive Faradaic efficiency of 90.1%. Our findings highlight how the deliberate spatial arrangement and chemical integration of distinct functional layers can unlock superior electrocatalytic behavior, offering design insights for next-generation water-splitting systems.

## Graphical abstract

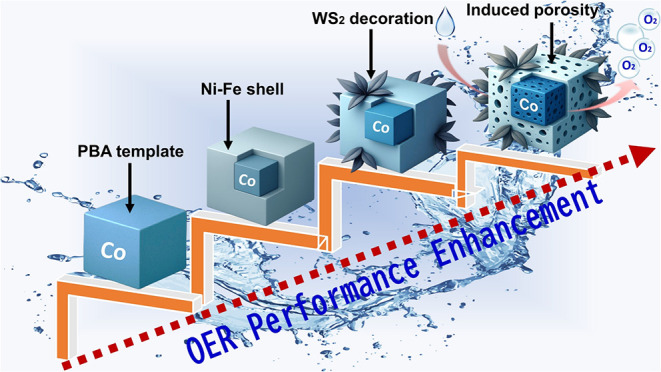

## 1 Introduction

Electrochemical water splitting (EWS) has emerged as a promising approach for producing clean hydrogen.^1^ However, the EWS efficiency is significantly hindered by the sluggish kinetics of the oxygen evolution reaction (OER), involving complex multielectron and proton-coupled steps. Although noble metal-based catalysts such as IrO_2_ and RuO_2_ exhibit excellent OER activity, particularly in acidic environments,^2,3^ their high cost and scarcity limit their practical deployment. Moreover, they are not ideal for EWS in neutral or alkaline media, where their performance advantage diminishes, and earth-abundant alternatives offer comparable activity with better cost-effectiveness and long-term stability.^4^ In this context, improving the electrochemical OER performance of transition metal (TM)-based catalysts is critical—if the high activation energy barrier is adequately lowered, it can significantly boost EWS reaction efficacy.^5^

Prussian blue analog (PBA) nanocubes, owing to their structural tunability and compositional flexibility,^6^ provide a versatile platform for incorporating multiple TMs that can work synergistically to enhance catalytic performance.^7^ However, pristine PBAs typically exhibit poor intrinsic activity due to their limited electrical conductivity and low density of catalytically active sites.^8^ Addressing this, engineered well-structured PBA nanocubes comprising Ni, Co, and Fe show potential to catalyze the OER effectively.^6,9,10^ The multivalent states of these TM ions boost the reaction rate by interacting with the oxygen intermediate, leading to bond formation through valence state changes.^6^ The intermediates formed in the OER process, such as M=O and M–O–O–, are crucial. The M–O bond in the M–O–O intermediate of the Co species can easily break and accelerate the oxygen release.^11^ The Fe species improves the electrode’s conductivity,^12,13^ or acts as an electron promoter itself, enabling high catalytic activity.^14^ It can also promote the formation of M=O due to the efficient charge transfer between the Fe and the Co or Ni atoms. Fe in the Co and Ni-based systems can modify the surface, which is beneficial for lowering the overpotential under alkaline conditions.^15,16^ The above facts highlight the need for multicomponent catalysts in which synergism enhances OER activity; however, decoupling the contributions of individual components and understanding their roles in active-site reconfiguration during OER remain black boxes in many cases. Besides that, efforts to improve the intrinsic activity of PBA nanocubes remain a challenge, as synthesizing well-defined multicomponent catalysts (core–shell, alloyed, layered, and single-atom sites) remains difficult, with uncertainty in precisely controlling their shape and size and in effectively regulating their electronic structure to improve electrocatalytic performance.^17^

Tenne’s pioneering work on transition-metal dichalcogenides (TMDCs) establishes these layered materials as attractive candidates for electrocatalysis. Their van der Waals interactions enable exfoliation of bulk crystals into monolayers, thereby increasing the density of exposed active sites.^18,19^ Among them, sulfide-based TMDCs are of particular interest because sulfur (S) anions can effectively modulate the coordination environment of TMs, thereby optimizing the adsorption energies of oxygenated intermediates involved in OER.^20^ S-anion-containing TMDCs exhibit enhanced intrinsic electrical conductivity and expose abundant active edge sites. Moreover, the flexible chemistry of TMDCs enables controlled anion substitution,^21^ making them ideal hosts for integrating S with other heteroanions such as phosphorus (P). This adaptability positions TMDCs as a robust foundation for constructing dual anion-coordinated M–S–P heterojunctions, unlocking superior catalytic performance.^22^

To harness the full strengths of each component, we adopted a step-by-step strategy to integrate them into a high-performance OER catalyst, in which each building block was introduced to counterbalance the inherent electrochemical limitations of individual components (e.g., limited active-site utilization and low intrinsic conductivity) and progressively enhance catalytic performance. In contrast to one pot synthesis of high-entropy multimetallic single-cube PBAs,^10,23^ where incorporating multiple metal species often leads to random distribution, phase complexity, and partial exposure of active sites, the step-by-step construction of a core–shell structure provides better control over morphology, avoiding random distribution of metals, and structural collapse due to nonuniform lattice strain from multiple metal incorporation under electrochemical stress. First, Co–Co PBA nanocubes provide a highly crystalline framework with well-defined morphology and abundant metal coordination sites, making them an ideal template for further engineering. To incorporate additional redox-active centers, we capped these with a Ni–Fe PBA shell, forming Co–Co@Ni-Fe PBA nanoframes, broadening the catalyst’s redox window. To further boost surface reactivity and electrical conductivity in the multimetallic backbone, we introduced exfoliated WS_2_ nanostructures onto the nanoframes. While bulk WS_2_ is catalytically inert for OER due to limited active edge sites and poor conductivity, anchoring it onto a metal-rich scaffold creates interfacial heterojunctions, facilitating electronic coupling, promoting faster charge transfer, and intermediate adsorption.^24^ The final step—gas-phase phosphidation—served multiple functions. The incorporation of P anion reconstructs the PBA into a defect-rich, conductive framework through structural etching, while preserving the PBA framework decorated with WS_2_, enabling efficient electrolyte penetration and rapid desorption of evolved oxygen bubbles. This positions the low-temperature phosphidation process as a deliberate design strategy rather than a mere post-treatment. The present work highlights the importance of rationally integrating structural and compositional features to overcome the intrinsic disadvantages of isolated constituents and achieve improved OER performance through cooperative effects, enabled by heterointerface M–S–P engineering. Going beyond, the resulting hybrid catalyst exemplifies a modular design strategy for next-generation electrocatalysts tailored for sustainable energy applications.

## 2 Experimental Section

### 2.1 Chemicals

Ammonium tetrathiotungstate [(NH_4_)_2_WS_4_] was obtained from Loba Chemie. Trisodium citrate dihydrate (C_6_H_5_Na_3_O_7_·2H_2_O), potassium ferrocyanide K_3_[Fe­(CN)_6_], potassium hexacyanocobaltate K_3_[Co­(CN)_6_], sodium hypophosphite monohydrate (NaH_2_PO_2_·H_2_0), cobalt nitrate hexahydrate (Co­(NO_3_)_2_·6H_2_O), nickel nitrate hexahydrate (Ni­(NO_3_)_2_·6H_2_0), poly­(vinylpyrrolidone) (PVP), and 5 wt % Nafion were purchased from Alfa Aesar. Dimethylformamide (HCON­(CH_3_)_2_) was purchased from Sigma-Aldrich. All chemical reagents were of analytical grade and were used as received without further purification. All electrolyte solutions were prepared with Milli-Q ultrapure water (>15 MΩ·cm).

### 2.2 Material Synthesis

#### 2.2.1 Synthesis of Co–Co PBA Nanocubes

The Co–Co PBA nanocubes were synthesized by the coprecipitation method.^25^ Typical amounts for the synthesis are solution A was prepared by mixing 0.6 mmol of Co­(NO_3_)_2_·6H_2_0 and 1.34 mmol of sodium citrate in 20 mL of deionized (DI) water. Solution B was prepared by dissolving 0.4 mmol of K_3_[Co­(CN)_6_] in 20 mL of DI water. Solution B was added to solution A under magnetic stirring for 15 min to uniformly disperse the particles. The resulting mixed solution was aged for 24 h at room temperature. The pink-colored product was collected by centrifugation, washed thrice with DI water and absolute ethanol, and dried at 60 °C overnight.

#### 2.2.2 Synthesis of Co–Co@Ni-Fe Core–Shell PBA Nanoframes

For the synthesis of Co–Co@Ni-Fe core–shell PBA nanocubes, 20 mg of the obtained Co–Co PBA nanocubes, 142 mg (0.49 mmol) of Ni­(NO_3_)_2_·6H_2_0, 0.25 mg of sodium citrate, and 0.3 g of PVP (K30) were dissolved in 30 mL of DI water to form a transparent green solution. Meanwhile, 66 mg (0.20 mmol) of K_3_[Fe­(CN)_6_] was dissolved in 20 mL of DI water to form solution B. Solution B was slowly added to solution A under magnetic stirring. After 15 min of stirring, the solution was aged for 24 h at room temperature without interruption. The resulting yellow precipitate was collected by centrifugation, washed thrice with DI water and absolute ethanol, and dried at 60 °C overnight.

#### 2.2.3 Synthesis of Co–Co@Ni-Fe PBA@WS_2_ Composites

For the synthesis of Co–Co@Ni-Fe PBA@WS_2_, 60 mg of Co–Co@Ni-Fe PBA and 20 mg of (NH_4_)_2_WS_4_ were dissolved in a 25 mL DMF solution. It was then sonicated for 15 min and heated for 10 h at 180 °C in a hydrothermal reaction. Post-treatment was done in the same manner to obtain a black-colored product.

The synthesis process of Co–Co@Ni-Fe PBA@S was similar to the above method except for replacing (NH_4_)_2_WS_4_ with thioacetamide.

As a control sample, WS_2_ nanoflowers were also synthesized similarly by dissolving 20 mg of (NH_4_)_2_WS_4_ in 25 mL of DMF and subjecting the mixture to hydrothermal reaction under the same conditions.

#### 2.2.4 Synthesis of Co–Co@Ni-Fe PBA@WS2-P Phosphidated Porous Nanocubes

The as-prepared Co–Co@Ni-Fe PBA@WS_2_ were further phosphidated in a tubular furnace using 20 mg of Co–Co@Ni-Fe PBA@WS_2_ and 200 mg of NaH_2_PO_2_. The two samples were placed at two different positions in a porcelain boat containing NaH_2_PO_2_ powder at the upstream side of the tubular furnace. The sample was annealed under an argon atmosphere at 350 °C for 2, 4, and 6 h, with a ramping rate of 2 °C min^–1^ to optimize the phosphidation time for the best catalytic activity.

As a control sample, the Co–Co@Ni-Fe PBA-P was prepared under identical conditions, except that the starting precursor was Co–Co@Ni-Fe PBA.

#### 2.2.5 Optimization of Phosphidation Duration

To identify the optimal phosphidation time, Co–Co@Ni-Fe PBA@WS_2_ composites were treated at 350 °C for 2, 4, and 6 h using NaH_2_PO_2_ as the phosphorus source under an argon atmosphere. Morphological evolution was tracked via FE-SEM imaging (Figure S2). The 2-h treatment induced only mild surface changes (Figure S2a–c), whereas extended phosphidation for 6 h led to structural damage and partial disintegration of the cubes (Figure S2g–i). The 4-h condition was optimal, maintaining overall cubic integrity while introducing edge roughness and porosity (Figure S2d–f). These features contributed to enhanced electrochemical performance and were thus chosen for all subsequent experiments.

### 2.3 Preparation of the Working Electrode

Each catalyst ink for the working electrode was prepared by dispersing 2 mg of the sample in a mixture of 200 μL water, 200 μL isopropyl alcohol, and 10 μL of 5 wt % Nafion binder, followed by ultrasonication for 15 min. Before using the glassy carbon electrode (GCE), the surface was polished with 0.3 and 0.05 μm alumina powders to smooth it. It was sonicated in water for 1 min in an ultrasonic bath to remove any particles, and then 30 CV cycles were performed in 0.50 M H_2_SO_4_ to clean it electrochemically. Finally, 10 μL of the ink (catalyst loading ∼0.69 mg cm^–2^) was drop-casted onto the thoroughly cleaned 3 mm GCE using a micropipette and dried at room temperature.

### 2.4 Characterizations and Electrochemical Measurements

The material characterizations and electrochemical assessment techniques are described in the Supporting Information.

## 3 Results and Discussion

### 3.1 Structural Characterization

Scheme 1 illustrates the step-by-step design approach for fabricating phosphidated trimetallic core–shell nanocubes adorned with WS_2_ nanostructures, thereby creating M–S–P heterojunctions. A simple coprecipitation approach generated homogeneous PBA nanocubes in an aqueous solution.^26^ Citrate ions participated in this process, controlling crystal nucleation and growth.^27^ A thin shell of Ni–Fe PBA was grown over the Co–Co PBA core to construct the core–shell nanoframes. Multiple metals in a core–shell configuration introduce redox diversity, enabling cooperative electron transfer and distributing the oxidative burden across neighboring sites.^6^ The nanoframes were decorated with WS_2_ nanostructures and phosphidated to generate an electronically coupled heterointerface in Co–Co@Ni-Fe PBA@WS_2_–P porous nanocubes.

**Scheme 1:**
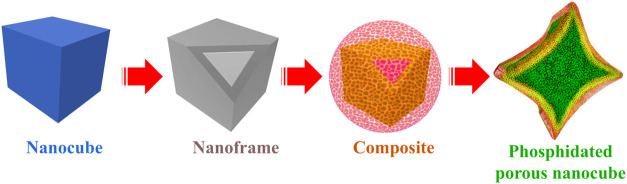
Morphological Evolution of Co–Co@Ni-Fe PBA@WS_2_-P Phosphidated Porous Nanocubes

In-depth microscopic analyses were done to understand the morphological evolution of the as-prepared catalysts. The field-emission scanning electron microscopy (FE-SEM) images inferred homogeneous Co–Co PBA nanocubes with a smooth surface and pointed edges and corners (facial length of 380 nm), as shown in Figure 1a. Transmission electron microscopy (TEM) images emphasized the thick, solid texture of the nanocubes with no apparent porosity (Figure 1b). A Ni–Fe PBA layer was grown at room temperature over Co–Co PBA nanocubes via epitaxial deposition, facilitated by their same crystal structure and lattice constant (Figure 1c).^25^ The TEM image with contrast differences showcased a uniform coating of Ni–Fe PBA on Co–Co PBA, creating a smooth shell with a thickness of 30.3 nm (Figure 1d). High-resolution TEM (HR-TEM) images of the nanoparticle’s corner region at different magnifications are shown in Figure S1.

**Figure 1:**
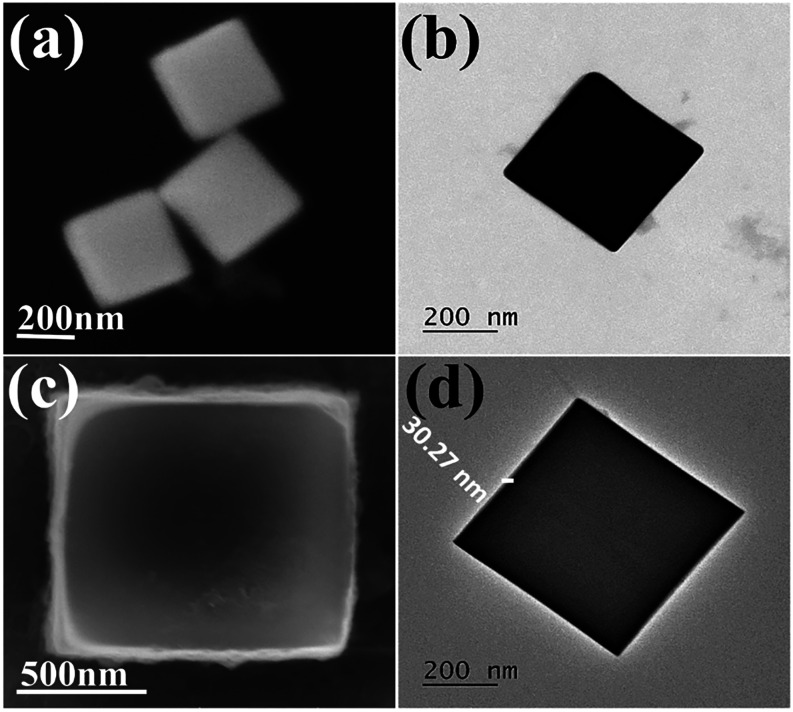
(a) FE-SEM image and (b) TEM image of Co–Co PBA (nanocubes); (c) FE-SEM image and (d) TEM image of Co–Co@Ni-Fe core–shell PBA (nanoframes).

The nanoframes underwent a hydrothermal treatment for 10 h at 180 °C, utilizing (NH_4_)_2_WS_4_ as the precursor for WS_2_. Dimethylformamide (DMF) served as the reducing agent to reduce W^VI^ to W^IV^.^16^ FE-SEM images showed the uniform growth of WS_2_ nanostructures across the entire nanoframe surface, resembling woolen balls (Figure 2a,b). TEM images confirmed the preserved cubic morphology of the core–shell PBAs, with the WS_2_ nanostructures wrapping around the edges and surfaces of the nanoframe (Figure 2c). Importantly, it highlighted the disparity in density between the PBA cubic frame and the woolly WS_2_ nanostructures, as the nanoframe, which was not visible under SEM (Figure 2b), is clearly depicted via TEM due to its more energetic, penetrating electron beam.

**Figure 2:**
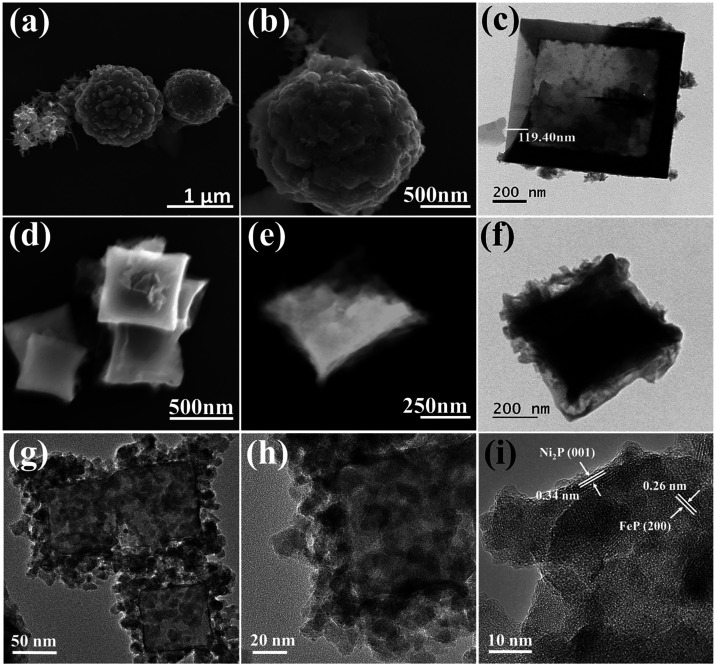
(a, b) FE-SEM images and (c) TEM image of Co–Co@Ni-Fe PBA@WS_2_ composites; (d, e) FE-SEM images and (f) TEM image of Co–Co@Ni-Fe PBA@WS_2_–P phosphidated porous nanocubes. (g, h) HR-TEM images show densely packed nanoparticles assembled across the cube surface; (i) high-resolution lattice fringes identifying FeP and Ni_2_P domains.

To enhance porosity, Co–Co@Ni-Fe PBA@WS_2_ composites were phosphidated at 350 °C for 2, 4, and 6 h, with the 4-h duration identified as optimal based on morphology (Figure S2) and electrochemical performance (see later discussion and Figure S10c). The resulting Co–Co@Ni-Fe PBA@WS_2_–P nanocubes retained their cubic shape with slight edge shrinkage, attributed to the higher reactivity of rough edges with PH_3_ and the elevated surface energy and unsaturated coordination at corners and edges.^28,29^ Detailed morphological characterization is presented in Figures 2d–f and S2. The HRTEM images of the Co–Co@Ni–Fe PBA@WS_2_–P sample show well-defined outlines of PBA cubes (Figure 2g) with the edges decorated by much smaller grains (Figure 2h). Higher magnification reveals these aggregates with lattice fringe spacings of 0.34 and 0.26 nm, corresponding to the (001) and (200) planes of Ni_2_P and FeP, respectively (Figure 2i).^30,31^

As a control, bare WS_2_ nanostructures were also synthesized via the hydrothermal treatment of (NH_4_)_2_WS_4_. The FE-SEM images showed WS_2_ nanostructures composed of aggregated nanosheets (Figure S3a,b). The dark gray TEM image of bulk WS_2_ suggested bulk thickness or stacking in the middle, while the edges appeared thinner (Figure S3c). The HR-TEM image revealed lattice fringes with an enlarged *d*-spacing (*d*_002_ = 0.68 nm), indicating exfoliation or interlayer expansion due to the intercalation of DMF solvent between the layers.

Elemental mapping using energy-dispersive X-ray spectroscopy (EDX) confirmed the core–shell structure of the Co–Co@Ni–Fe PBA nanoframe. Figure S4 portrays the Co-rich core, with Ni and Fe primarily distributed along the outer edges and corners, indicating compositional heterogeneity. EDX quantification shows a Ni/Fe atomic ratio of 1.2, with a slightly higher Ni content (Table S1). Since Co formed the core, it accounted for approximately 80% of the overall elemental composition. STEM-EDX mapping of the WS_2_ wrapped composite revealed a uniform distribution of W and S across the nanoframe, along with Ni and Fe on the outer surface (Figure 3a). The Ni/Fe ratio remained consistent at ∼1.2, with a dominant Co signal attributed to the core (Table S1).

**Figure 3:**
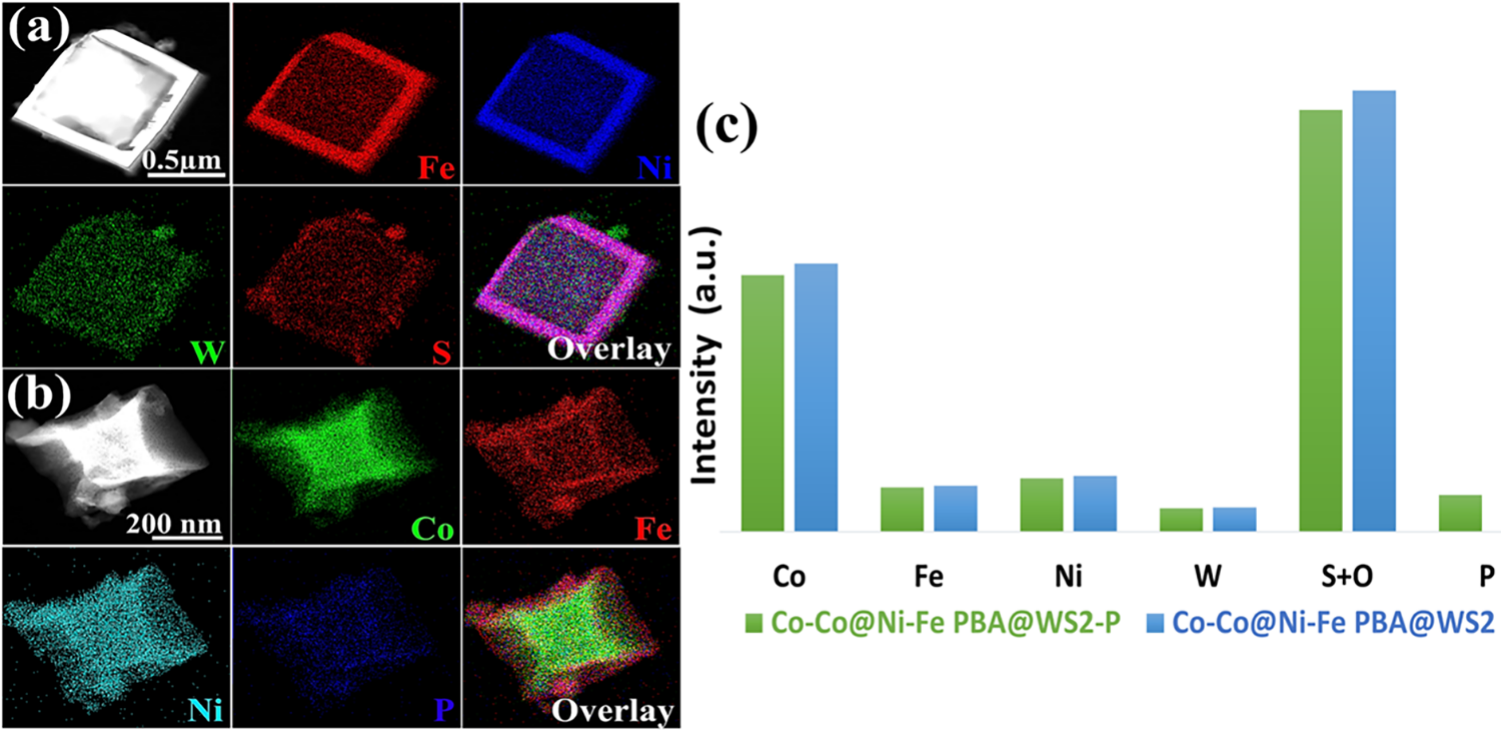
HAADF-STEM images of (a) Co–Co@Ni–Fe PBA@WS_2_ and (b) Co–Co@Ni–Fe PBA@WS_2_–P, with corresponding elemental mappings showing the distribution of Fe, Ni, W, S, and Co, Fe, Ni, and P along with their overlays. (c) EDX intensity profiles comparing the elemental compositions of Co–Co@Ni–Fe PBA@WS_2_–P and Co–Co@Ni–Fe PBA@WS_2_.

In the phosphidated porous nanocubes, STEM-EDX confirmed the presence of Co, Ni, Fe, and P (Figure 3b). At the same time, separate elemental maps in Figure S5 demonstrated the homogeneous distribution of W, S, and P throughout the structure. Notably, after phosphidation, the Ni/Fe ratio remained unchanged, and Co remained the primary component. However, the atomic percentages of each element slightly decreased compared to the Co–Co@Ni–Fe PBA@WS_2_ composite. The total reduction in elemental composition was about 4.3%, aligning well with the 4.4% P incorporated into the catalyst (Table S1). Notably, Figure 3c compares the elemental transformations during phosphidation, showing that the combined intensity of S + O decreases with the introduction of P. At the same time, the TM framework (Co, Ni, Fe, W) remains largely unaffected. The observed reduction in S and O content after phosphidation suggests a partial replacement of these anions by P, leading to the formation of M–P and M–S–P heterojunctions, as evidenced by lattice fringes and subsequent XRD and XPS analyses.

Additionally, it is possible that weakly bound oxygenated species, originating from adsorbed water or surface oxidation, are removed under the phosphidation conditions. The inductively coupled plasma (ICP) measurements of all the as-synthesized catalysts further confirmed that the Ni/Fe atomic ratio is approximately 1.5, in line with the theoretical ratio (3:2) and the EDX elemental analysis (1.2). The results were summarized in Table S2.

Powder X-ray diffraction (XRD) analysis confirmed the phase purity of the synthesized catalysts. The highly crystalline nature of the Co–Co PBA template was confirmed by the sharp reflections within the 2θ range of 5°–80°, as shown in Figure 4a. The sharp peaks for (200), (220), (400), (331), (420), and (422) planes suggested the excellent crystallinity of the synthesized nanocubes, indexed to cubic Co_3_[Co­(CN)_6_]_2_ (JCPDS no. 77–1161).^6,32^ Table S3 presents the calculated relative crystallinity based on integrated XRD intensity. No additional peaks were detected, indicating the product’s purity. Notably, the PBAs are known to be nonstoichiometric, typically containing variable amounts of [Co­(CN)_6_]^4–^ vacancies in Co–Co PBA and lattice water,^33,34^ which can modify the scattering power of specific crystallographic planes. Such structural variation causes the (420) reflections to appear with disproportionately high intensity compared to the standard JCPDS pattern. The diffraction peaks of Co–Co@Ni-Fe PBA core–shell nanoframes stemmed from Co–Co PBA and Ni–Fe PBA (JCPDS no. 46–0906), both having similar crystallographic features, as reported before.^25^ However, upon shell formation, the peaks shifted slightly to lower angles, suggesting an increase in *d*-spacing due to local strain or lattice mismatch introduced by the shell.^35,36^

**Figure 4:**
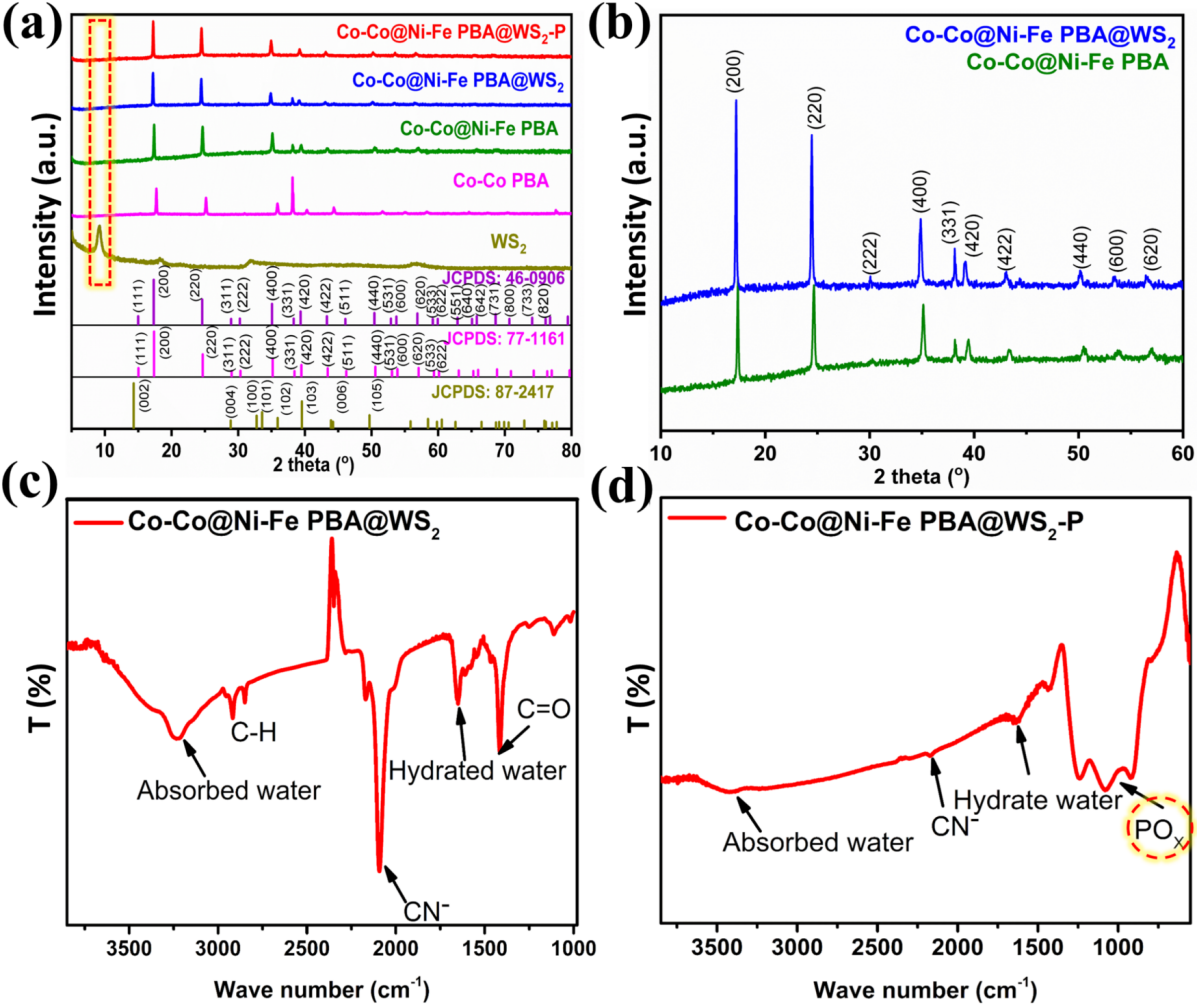
(a) XRD pattern of WS_2_ nanostructures, Co–Co PBA nanocubes, Co–Co@Ni-Fe PBA nanoframes, Co–Co@Ni-Fe PBA@WS_2_ composites, and Co–Co@Ni-Fe PBA@WS_2_–P phosphidated porous nanocubes; (b) Zoom-in XRD plot of Co–Co@Ni-Fe PBA nanoframes and Co–Co@Ni–Fe PBA@WS_2_ composites. (c-d) FTIR spectra of the Co–Co@Ni-Fe PBA@WS_2_ composites and Co–Co@Ni-Fe PBA@WS_2_–P phosphidated porous nanocubes.

The XRD pattern of the as-synthesized Co–Co@Ni-Fe PBA@WS_2_ closely resembled Co–Co@Ni-Fe PBA. Upon WS_2_ growth, the zoomed-in XRD pattern of Co–Co@Ni-Fe PBA@WS_2_ also showed a slight shift of the peaks toward lower diffraction angles compared with Co–Co@Ni-Fe PBA (Figure 4b). Such an observation suggests that the two components—core–shell PBAs and WS_2_—are not physically attached but involve chemical interactions that form M-S bonds between Co/Ni/Fe and S, leading to heterojunction formation (also confirmed by XPS analysis, shown below). Notably, the sample does not exhibit the (002) peak of WS_2_, which typically corresponds to periodicity along the *c*-axis.^37^ The absence of (002) reflection is indicative of single-layer or a few-layer WS_2_ sheets.^37,38^ After phosphidation, the XRD pattern of the phosphidated porous nanocubes showed no new peaks; however, closer observations revealed that the peak at 38° is absent, which accounts for a phase evolution from a hydrated, vacancy-rich PBA structure to the M-P heterostructure. The observed 35.15°, 39.15°, and 53.14° reflections align well with the (112), (021), and (004) planes reported for the Ni_2_P phases (JCPDS 00–074–1385), indicating that the final material is partly converted to a catalytically active phosphide phase. The sample still retained high crystallinity (85.5%), slightly lower than that of its unmodified counterpart, likely due to defect formation during phosphidation (Table S3). The XRD patterns of bulk WS_2_ were also presented in Figure 4a. Compared with its typical peak values in the JCPDS no. 87–2417,^39^ the (002) diffraction peak of hexagonal WS_2_, usually located at 2θ of 14.4°,^40^ shifted to a lower angle of 9.0° due to the increase in interlayer spacing (as seen from the lattice fringe *d*-spacing value). This characteristic feature was attributed to the intercalation of the DMF solvent between the WS_2_ layers.^41^

Figure 4c,d presents the Fourier transform infrared (FTIR) spectra of nonphosphidated and phosphidated porous nanocubes. In the Co–Co@Ni–Fe PBA@WS_2_ composite spectrum, the peaks corresponding to C–H and C=O bonds originated from the DMF solvent (Figure 4c). However, in the phosphidated porous nanocubes (Figure 4d), these peaks disappeared as the solvent vaporized. Simultaneously, distinct peaks of PO_X_ emerged while the CN^–^ peak diminished. Additionally, heat treatment reduced the amount of water absorbed. Figure S6a,b presents the thermogravimetric analysis (TGA) plots of the composites and phosphidated porous nanocubes conducted under an air atmosphere. The TGA curve of the Co–Co@Ni–Fe PBA@WS_2_ composites displayed a weight loss in the temperature range from room temperature to 140 °C, which can be attributed to the evaporation of moisture. Subsequent weight loss corresponded to the complete decomposition of the cyanide bridges in the structure (Figure S6a), with a broad exothermic differential thermal analysis (DTA) peak (Figure S6a, **inset**).^25^ In contrast, the TGA curve of the phosphidated catalyst revealed decisive observations. A sharp weight loss associated with water desorption was initially monitored (Figure S6b). The DTA exotherm appears broader and less intense, as shown in Figure S6b (**inset**). Additionally, a lower percentage of catalyst weight loss was observed because the DMF remnants had already been removed during the phosphidation process. Notably, a weight gain was observed at higher temperatures, indicating the oxidation of the P species under these conditions. The corresponding DTA plot shows an exothermic peak in the same temperature range, consistent with oxidation being a heat-releasing reaction.

The surface chemical composition and elemental valence states of the Co–Co@Ni–Fe PBA@WS_2_ composites before and after phosphidation were systematically analyzed using X-ray photoelectron spectroscopy (XPS). The survey spectra (Figures S7 and S8a) confirmed the presence of Fe, Co, Ni, W, S, O, and, notably, P after phosphidation. The increased oxygen peak intensity postphosphidation correlated well with the FTIR and TGA results, indicating surface oxidation and formation of phosphate (PO_X_) species upon air exposure. The oxidation states and electronic environment of the elements in the Co–Co@Ni–Fe PBA@WS_2_ composites are shown in Figure S8b–f. Figure S9 presents the XPS data for Co–Co@Ni–Fe PBA-P; a detailed explanation is provided in the SI. In the Co 2p high-resolution XPS spectrum of the Co–Co@Ni–Fe PBA@WS_2_ composites and phosphidated catalyst, Figures S8b and 5a, the binding energy (BE) peaks centered at 783.5 and 799.0 eV were assigned to the spin orbitals of Co 2p_3/2_ and Co 2p_1/2_ in Co–O, which can be at both Co^2+^ or Co^3+^ oxidation states.^42^ Thus, the contact with oxygen in the air results in partial surface oxidation of the cobalt. The slight shift to lower BEs in the phosphidated sample (e.g., 783.3 to 783.5 eV and 798.8 to 799.0 eV) suggests a minor reduction of Co species. The consistency of these peaks near the characteristic BE values for Co^2+^ and Co^3+^ states in Co–O indicates that the core electronic structure of cobalt remains largely stable, with only slight surface modifications likely due to the addition of P atoms, probably because the Co is an inner element in the catalyst structures. The satellite peaks at ∼788.5 and ∼ 805 eV are characteristic of Co^2+^ and Co^3+^ in the high-spin state.^43^

**Figure 5:**
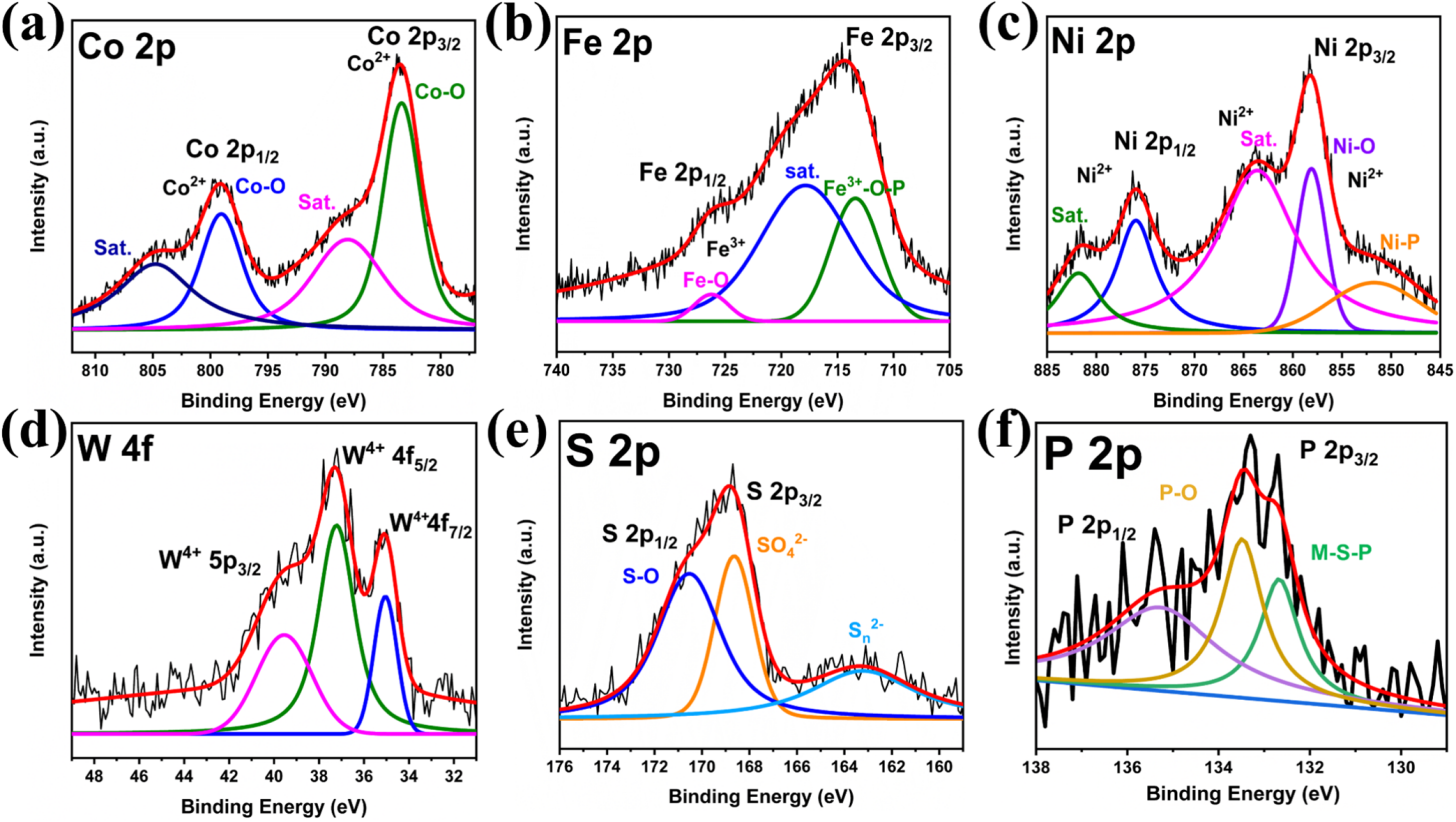
High-resolution XPS spectra of Co–Co@Ni-Fe PBA@WS_2_–P phosphidated porous nanocubes showing Co 2p (a), Fe 2p (b), Ni 2p (c), W 4f (d), S 2p (e), and P 2p (f).

In the Fe 2p spectrum (Figure 5b), the peak at 726.3 eV can be assigned to the Fe 2p_1/2_ orbital of a highly oxidized iron species, consistent with reports of Fe^3+^ in phosphorus-rich or oxide environments, which exhibit similar positively shifted binding energies (≈725–726 eV).^44^ The shift compared to the nonphosphidated reference (Figure S8c) at 722.6 eV suggests an increase in oxidation state or ligand electronegativity—likely due to bonding with P^δ−^ or O^δ−^—and supports partial electron withdrawal (M^δ+^–P^δ−^). The satellite-like feature at 717.6 eV corresponds to the typical Fe^3+^ shakeup signal, as documented in Fe-containing TM systems.^45^ The prominent peak at 713.3 eV is characteristic of Fe 2p_3/2_ in oxidized iron bound to electronegative ligands (such as P or O), located between known ranges for Fe^3+^–S (709.5–711.0 eV) and Fe^3+^–O (710.3–712.9 eV), suggesting mixed Fe–O–P coordination following phosphidation. These spectral assignments collectively indicate that phosphidation induces the formation of Fe–P and Fe–O species with an increased iron oxidation state, while ruling out the generation of new Fe–S bonds.

In Figure 5c, the Ni 2p spectrum of the phosphidated sample shows a peak at 851.4 eV, which can be attributed to Ni^δ+^ in a phosphidic environment (Ni–P). The broader shift from typical metallic Ni values supports the presence of Ni–P bonding. The peak at 858.0 eV corresponds to Ni^2+^ in Ni–O and remains unchanged relative to the nonphosphidated sample (Figure S8d), suggesting the persistence of nickel oxide species even after phosphidation.^46^ The satellite peaks observed at 863.6 and 881.7 eV are characteristic of Ni^2+^ shakeup features, supporting the continued presence of oxidized Ni states. Altogether, the spectra indicate the coexistence of both oxidized and phosphidic nickel environments following phosphidation. The W 5p_3/2_, W 4f_5/2_, and W 4f_7/2_ XPS peaks of the phosphidated porous nanocubes were located at 39.6, 37.2, and 35.0 eV, respectively, confirming the presence of W^4+^ (Figure 5d).^47^ Similar peaks at lower BE appeared in the case of the Co–Co@Ni–Fe PBA@WS_2_ composites at 37.6, 35.3, and 32.3 eV, respectively (Figure S8e),^47^ indicating a more oxidized chemical environment following phosphidation. The peak positions correspond to a trigonal prismatic configuration of W atoms and are identical to those of the thermodynamically stable semiconducting 2H-phase WS_2_.^47^ Such shifts suggest partial electron withdrawal from tungsten atoms, consistent with the incorporation of more electronegative ligands such as phosphorus or oxygen.

In Figure 5e, the S 2p_3/2_ peak at 163.2 eV in the phosphidated porous nanocubes corresponds to S_n_^2–^ species, associated with metal–sulfur bonds.^48^ In the nonphosphidated sample (Figure S8f), the peak at 162.2 eV also indicates M–S coordination, typical of WS_2_.^47^ Upon phosphidation, the emergence of a peak at 168.7 eV reflects the formation of oxidized sulfur species, notably SO_4_^2–^. Additionally, the shift of the oxidized sulfur peak from 169.2 to 170.5 eV further confirms sulfur oxidation induced by phosphidation, creating an electron–proton mediation environment, where S–O_*x*_ species balance charge at the interface and lower the kinetic barrier for O–O bond formation, directly contributing to enhanced OER activity. These shifts support the successful chemical transformation of Co–Co@Ni-Fe PBA@WS_2_ to Co–Co@Ni-Fe PBA@WS_2_–P via a low-temperature gas phosphidation process. In Figure 5f, the peak at 133.3 eV was attributed to oxidized phosphorus species (e.g., P–O, P=O), likely due to surface exposure to air after phosphidation.^49^ The peak at 132.6 eV is consistent with M–S–P bonding (shifted from 128.8 eV in the presence of WS_2_ layering, as seen in Co–Co@Ni-Fe PBA-P, Figure S9e).^50^ It should be emphasized that while HRTEM lattice fringes and XRD primarily reflect the bulk M–P phase, surface-sensitive XPS analysis indicates the formation of an M–S–P heterojunction (thiophosphate-like motifs) at the material surface, suggesting phase differentiation between the bulk and surface regions. Positive shifts in BE for Fe 2p, Ni 2p, S 2p, and W 4f following phosphidation indicated increased oxidation and electron withdrawal, consistent with M–S–P bond formation. The Co 2p peak position remained unchanged, suggesting its oxidation state was unaffected, possibly due to its location in a more internally shielded region of the catalyst. The observed charge redistribution, with electron-rich P and oxidized metals, supports improved M-to-P electron transfer and highlights the phosphidated porous nanocubes as promising OER electrocatalysts. Table S4 summarizes the elemental quantification determined from XPS analysis.

### 3.2 Electrochemical OER Performance

The OER performance of the as-prepared catalysts was evaluated at 1.0 M KOH using a standard three-electrode setup. The Co–Co@Ni–Fe PBA@WS_2_–P phosphidated porous nanocubes exhibited the highest catalytic activity, among the tested samples, requiring an overpotential of only 280 mV at 10 mA cm^–2^ current density (η_10_) for water oxidation (Figure 6a). This performance is comparable to state-of-the-art Ni–Fe-based electrocatalysts^51^ and commercial RuO_2_ (312 mV @ 10 mA cm^–2^) or IrO_2_ (288 mV @ 10 mA cm^–2^).^52,53^ The highest OER performance was attributed to cooperative interactions among multiple metal centers and dual anions at the M–S–P interface. Specifically, Fe centers facilitate the formation of OOH intermediates and improve electronic conductivity.^54^ In parallel, Co and Ni sites promote O–O bond formation, which is essential for the rate-determining step of the OER cycle.^37,55^ The incorporation of P and S anions further tunes the catalyst’s electronic structure, enhancing electrical conductivity and optimizing adsorption energies for key OER intermediates. At the M–S–P interface, new “balanced” catalytic sites emerge that cannot exist in the pure M–S (sites for OH* and OOH* adsorption) or pure M–P (site for accelerating *OH → *O and *O → *OOH steps) phases. Specifically, reported DFT calculations and XPS analyses of the S-doped NiFeP system showed that sulfur incorporation increases the oxidation states of Fe and Ni, redistributes electrons toward S and P atoms, and lowers the energy barrier for OOH* formation—the rate-determining step in the OER pathway.^56^ Thus, at the heterojunction, electron density redistributes across M, S, and P, creating a built-in polarity that optimizes intermediate BEs. This means that even M–S or M–P individually perform well in OER, the heterojunction combines the strengths of both: S anion improves the adsorption kinetics by donating electrons, which stabilize OH* and OOH* intermediates and accelerates the initial adsorption steps; P anion withdraws electrons, preventing O* from overbinding on the M centers, ensuring efficient O_2_ release, and finally, the TMs stabilize the framework and enable formation of high-valent M–O intermediates through multinuclear O–O coupling.

**Figure 6:**
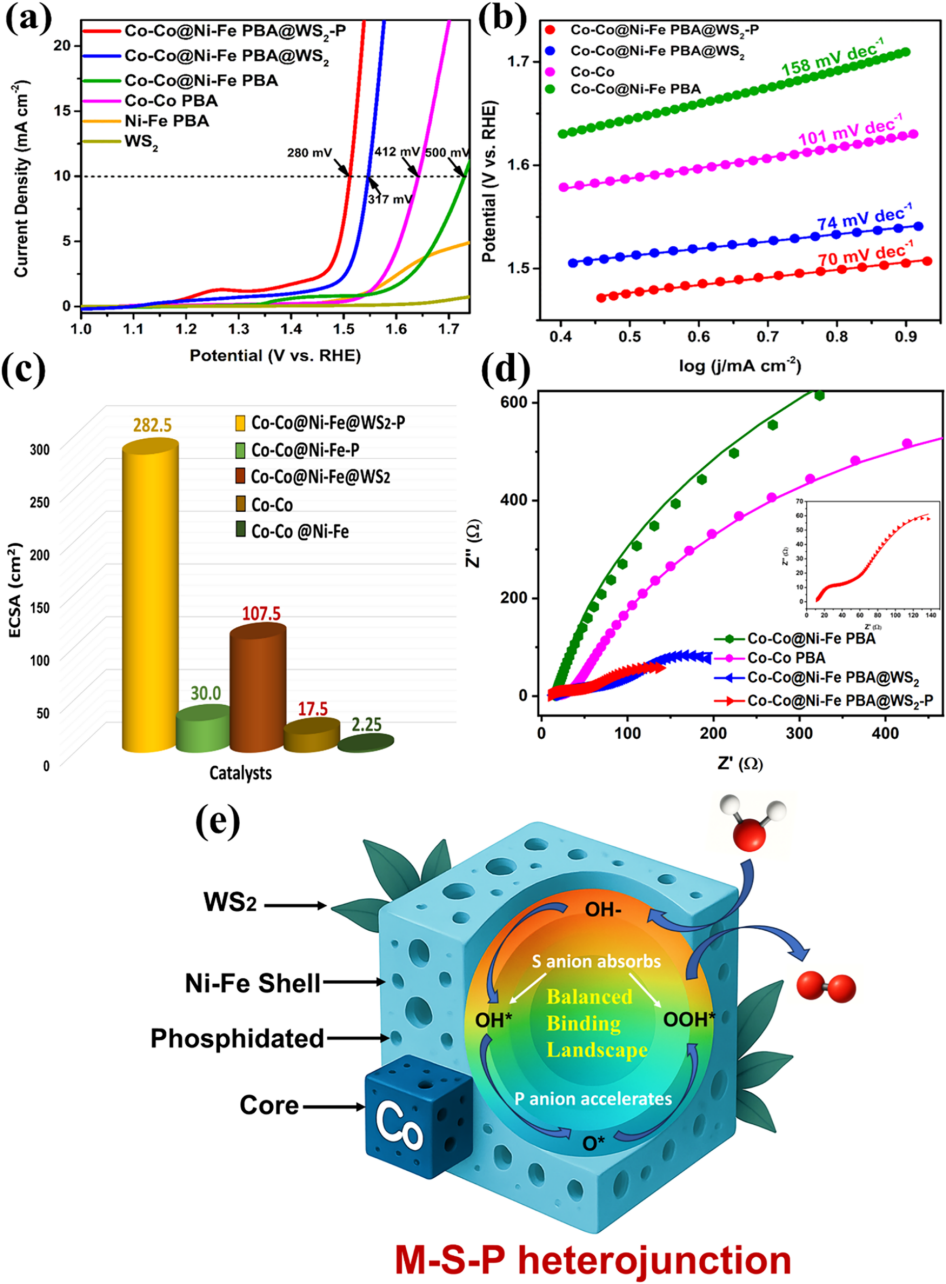
(a) LSV curves in 1.0 M KOH solution; (b) Corresponding Tafel plots; (c) ECSA comparison of different catalysts; (d) EIS-Nyquist plots measured at 1.51 V vs RHE; the inset shows the zoom-in plot of Co–Co@Ni-Fe PBA@WS_2_–P phosphidated porous nanocubes; (e) Illustration of the complementary roles of dual anions in the M–S–P heterojunction.

In contrast, the Co–Co@Ni–Fe PBA@WS_2_ composites, Co–Co PBA nanocubes, and Co–Co@Ni–Fe PBA nanoframes achieved the benchmark current density η_10_ at overpotentials of 317, 412, and 500 mV, respectively. Meanwhile, the Ni–Fe PBA and bulk WS_2_ nanostructures could not reach 10 mA cm^–2^ even at 1.75 V vs RHE, indicating their inherently low catalytic activities. From this observation, we can safely conclude that, although some of the best-performing OER catalysts are Ni–Fe layered double hydroxides with a strong interaction between Ni and Fe,^57,58^ in PBAs, this interaction does not always translate into a rigid, insulating PBA framework. On the other hand, the activity of Co–Co PBA does not rely on such interaction—it can drive OER independently with high intrinsic activity. The poor performance of pristine WS_2_ is attributed to its relatively low electrical conductivity. For comparison, the LSV curves of Co–Co@Ni–Fe PBA@S and Co–Co@Ni–Fe PBA@WS_2_ are shown in Figure S10a, highlighting the cooperative role of W^4+^ and S^2–^ in enhancing performance. While sulfur atoms aid in immobilizing active species and improving catalytic response, W^4+^ ions facilitate charge transfer and modulate the local electronic environment, thereby optimizing the adsorption of OER intermediates and enhancing the intrinsic electrocatalytic activity.^59^

Similarly, the OER performance of the control sample Co–Co@Ni–Fe PBA–P was evaluated under the same conditions, delivering an overpotential (η_10_) of 300 mV, higher than the phosphidated porous nanocubes but lower than the nonphosphidated ones (Figure S10b). This result supports the beneficial effect of phosphidation in enhancing surface permeability and exposing more electrocatalytically active sites.^60^ It can be explained as when OER proceeds via *OH deprotonation to form *O, or when OH^–^ attacks *O to form *OOH, steps requiring fast proton-coupled electron transfer, the P anion stemming from the M-P system acts as a local proton relay, transiently capturing the proton from the adsorbate, and then releasing it to the electrolyte, which in turn shortens the proton-transfer distance, lowers reorganization energy, and stabilizes the transition state, preventing the *OH → *O and *O → *OOH conversions from becoming rate-limiting steps. In the fully assembled Co–Co@Ni–Fe PBA@WS_2_–P system, which exhibited a lower overpotential, experiences a strong influence of dual anions. While mixed-metal catalysts generally outperform single-metal systems due to improved charge transfer and electronic modulation,^61^ the incorporation of a Ni–Fe PBA shell over a Co–Co core initially reduced activity, likely due to the lower intrinsic activity of Ni and Fe compared to Co. Co-based catalysts, especially those involving Co^4+^ (from the CoO_2_/CoOOH redox pair), exhibit favorable *OH adsorption and faster subsequent OER steps than Ni-based systems.^62^ Nevertheless, the phosphidated Ni–Fe shell contributes to higher performance because the phosphidated Ni–Fe shell directly translates into the formation of actual OER-active sites at the surface, where the synergy of P-mediated proton transfer and M-induced electronic tuning lowers kinetic barriers and boosts overall catalytic activity, while the unmodified Co–Co PBA core remains primarily a conductive backbone. Additionally, the isotropic structure of M–P features more surface-unsaturated coordination atoms than that of M–S. Consequently, M–P exhibits higher catalytic activity.^63^ Among various phosphidation durations, the sample treated for 4 h showed the highest activity (Figure S10c), suggesting an optimal balance between structural transformation and retention of porosity and morphology.

Tafel plots, derived from the polarization curves (Figure 6a), provided insight into the OER kinetics. The phosphidated porous nanocubes exhibited the lowest Tafel slope of 70 mV dec^–1^, outperforming the Co–Co@Ni–Fe PBA@WS_2_ composites (74 mV dec^–1^), Co–Co PBA (101 mV dec^–1^), and Co–Co@Ni–Fe PBA nanoframes (158 mV dec^–1^), Figure 6b. Lower Tafel slopes reflect faster charge-transfer kinetics, indicative of enhanced electrocatalytic performance.

To correlate this with the density of active sites, the electrochemical surface area (ECSA) was estimated via double-layer capacitance (*C*_dl_) derived from CV curves recorded between 1.02 and 1.12 V (vs RHE) in a nonfaradaic region (Figures S11a–d and S12a). As expected, the trend in *C*_dl_ values matched the order of activity. The phosphidated porous nanocubes exhibited the highest *C*_dl_ (11.3 mF cm^–2^), followed by Co–Co@Ni–Fe PBA@WS_2_ (4.3 mF cm^–2^), Co–Co@Ni–Fe PBA–P (1.2 mF cm^–2^), Co–Co PBA (0.7 mF cm^–2^), and Co–Co@Ni–Fe PBA nanoframes (0.09 mF cm^–2^), Figures S11e and S12b. ECSA was calculated using the equation ECSA = *C*_dl_/*C*_s_, where *C*_dl_ is the double-layer capacitance, and *C*_s_ is the specific capacitance. A general specific capacitance of 40 μF cm^–2^ (in 1.0 M KOH) was used, as reported previously, to calculate the ECSA values of all catalysts deposited on glassy carbon electrodes.^16,26^ These results confirm that Co–Co@Ni–Fe PBA@WS_2_–P exhibits the largest ECSA and the highest density of exposed catalytic sites, as shown in Figure 6c.

Notably, the suppressed activity in Co–Co@Ni–Fe nanoframes compared to Co–Co PBA suggests that shell growth may obscure electrochemically active Co sites. Phosphidation partially recovers this by increasing surface area and porosity; however, the most significant enhancement is achieved by incorporating WS_2_ nanostructures (Figure 6c). In Co–Co@Ni–Fe PBA@WS_2_, vertically aligned WS_2_ exposes more edge sites—known to be catalytically active—than the basal plane dominated in pristine WS_2_.^64^ The chemically coupled interface acts like an electron bridge^51^ allowing redox-active metal centers of the core–shell PBA to communicate effectively with the catalytically active WS_2_ edges. Thus, the superior OER performance of the phosphidated porous nanocubes stems from a combination of vertically grown WS_2_, which increases accessible active sites, and phosphidation, which enhances porosity and facilitates electrolyte penetration and gas evolution. Moreover, TMPs contribute hydrogenase-like activity due to the partial positive charge on the metal and the negative charge on phosphorus, enabling the simultaneous adsorption and activation of reaction species^65^

The high electronic conductivity of the phosphidated porous nanocubes ensured efficient electron and proton transport during the OER process. Electrochemical impedance spectroscopy (EIS) measurements, presented in Figure 6d, along with the corresponding Randles equivalent circuit (Figure S13), revealed that the phosphidated porous nanocubes exhibited the lowest charge-transfer resistance (*R*_ct_) of 46.4 Ω, significantly lower than those of the nonphosphidated nanocubes (140.3 Ω), Co–Co PBA (1312.5 Ω), and Co–Co@Ni–Fe PBA (2108.0 Ω), Table S5. The internal electric field at the heterojunction promotes directional electron flow from the sulfide-phosphide interface to the in situ formed oxide surface, reducing the *R*_ct_ value. WS_2_ nanostructures tend to orient vertically on nanostructured surfaces during hydrothermal growth. The vertically grown electron-rich S sites form a better electrical contact with the underlying PBA template, resulting in a considerable reduction in the *R*_ct_ value (favorable sites for O–O bond formation). However, the lowest *R*_ct_ value postphosphidation confirms superior conductivity from dual anions, providing kinetic acceleration that, along with the enlarged ECSA, explains the enhanced OER performance. The improved conductivity arises from the in situ formation of metal oxide/hydroxide species at the sulfide/phosphide heterointerface under electrochemical conditions. This surface reconstruction increases the electrochemically active surface area and facilitates charge transport, thereby enhancing electrochemical activity.^66,67^ Thus, it is essential to note that the as-synthesized catalyst does not represent the intrinsic active phase; instead, it undergoes in situ surface reconstruction that provides the actual catalytically active sites. On this basis, the catalyst is more accurately described as a ‘precatalyst’ rather than the true active catalyst.

Beyond catalytic activity, electrochemical durability is crucial for practical applications, particularly in multicomponent catalysts, which are prone to surface segregation or leaching, under harsh OER conditions, at high current densities, or in alkaline/acidic media. Chronoamperometric stability tests at constant current densities of 10 and 20 mA cm^–2^ showed negligible degradation over 24 h (Figures 7a, **inset** and S14), indicating excellent stability. Likewise, linear sweep voltammetry (LSV) curves recorded before and after 1000 cycles between 1.05 and 1.6 V vs RHE revealed minimal change in overpotential at 10 mA cm^–2^, further confirming the robust nature of the M–S–P heterojunction.

**Figure 7:**
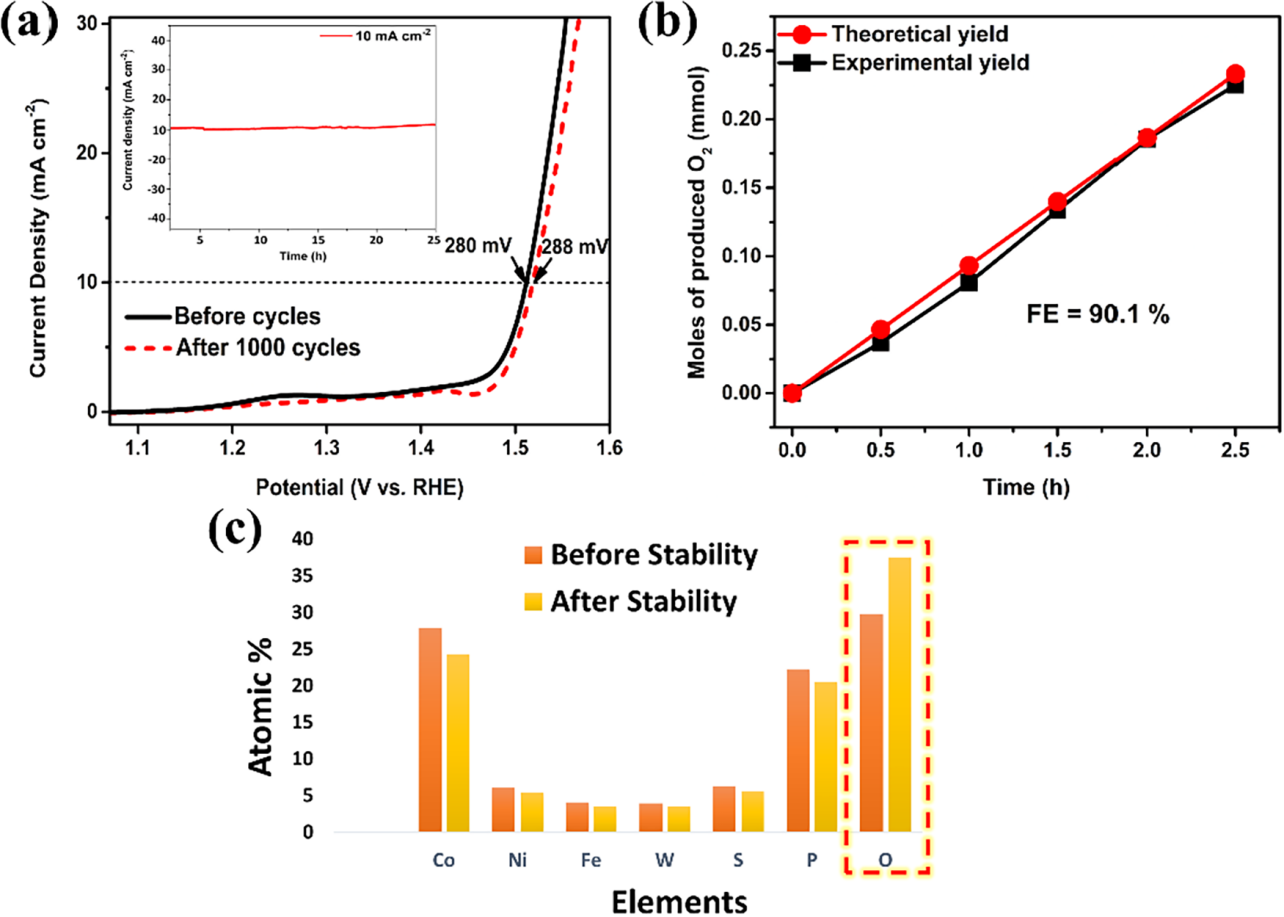
(a) OER stability test—LSV curves of the Co–Co@Ni-Fe PBA@WS_2_–P phosphidated porous nanocubes before (solid black) and after (dashed red) 1000 CV cycles, the inset shows the chrono-amperometric time-dependent current density curve during electrolysis at ∼1.51 V for 24 h on phosphidated porous nanocubes. (b) Comparison of theoretical and experimental O_2_ yields with a calculated faradaic efficiency of 90.1%. (c) Elemental composition (atom %) before and after stability test.

To assess the reaction selectivity, the amount of oxygen evolved at 10 mA cm^–2^ was measured and compared to the theoretical value to determine the Faradaic efficiency (FE). The phosphidated catalyst achieved an impressive FE of ∼90.1%, indicating that the majority of the observed current was effectively utilized for water oxidation (Figure 7b). This high FE demonstrates the catalyst’s capability to selectively and efficiently drive the OER process.

FESEM images taken after the catalytic tests (Figure S15) show that the phosphidated porous nanocubes largely retained their original cubic framework, though with noticeable deformation of the cubes. This structural change is attributed to the formation of an oxyhydroxide surface layer. Such amorphization is beneficial, as OER reactions generally proceed more efficiently on amorphous surfaces than on crystalline ones due to their higher electrochemically active surface area, increased density of defect sites, greater structural flexibility, and improved corrosion resistance.^68^ Unlike amorphous materials, crystalline materials often exhibit blocked active sites, limiting the effective contact area between the catalyst and the electrolyte.

Elemental analysis via EDX revealed an increased atomic percentage of oxygen after 1,000 OER cycles, accompanied by a decrease in all other elements, supporting the formation of a surface oxyhydroxide layer (Table S6 and Figure 7c). This in situ formation of oxyhydroxide layer (real active sites) plays a protective role by preserving the core–shell structure (there is no significant leaching of the TMs or loss of the heteroatom decoration) and preventing excessive oxidation of the inner disulfide core, thereby contributing to the long-term stability of the catalyst under OER conditions. Importantly, compared to other noble-metal-free sulfide and phosphide-based OER electrocatalysts reported in the literature, the dual-anion coordinated Co–Co@Ni–Fe PBA@WS_2_–P phosphidated porous nanocubes demonstrated a notably lower overpotential and Tafel slope (Table S7). The cooperative effect resulting from the coexistence of sulfide and phosphide domains establishes a highly active dual-anion coordination that optimizes the adsorption energies of oxygen-evolution intermediates (e.g., *OH, *O, *OOH), thereby enhancing the intrinsic catalytic activity in the M–S-P heterojunction, Figure 6e. Such a synergistic interplay is not observed in single-anion phosphidized or sulfurized PBAs, which explains their comparatively lower activity.

## 4 Conclusions

The study introduces a model system for designing hierarchical electrocatalyst architectures, demonstrating a clear relationship between structure and performance for efficient, durable oxygen evolution catalysis. The design sequence of integrating a multimetallic core–shell architecture with conductive, chemically tunable surface components yielded a porous framework with enhanced electrical conductivity, a high surface area, and numerous accessible active sites. Using these features, the Co–Co@Ni–Fe PBA@WS_2_–P catalyst achieves an OER performance with an overpotential of 280 mV at 10 mA cm^–2^, a Tafel slope of 70 mV dec^–1^, and approximately 90% Faradaic efficiency. The catalyst’s performance benefits from various structural and electronic factors: the Ni–Fe PBA shell supplies redox-active sites and stability; the vertically aligned WS_2_ improves charge transport and surface accessibility; and the cobalt-rich core, which has high intrinsic OER activity—while essentially embedded—remains partially accessible, as confirmed by XPS, contributing to the electronic modulation of the shell. The in situ formation of an oxyhydroxide surface layer (real active sites) during operation, especially involving Co and Fe atoms, helps stabilize the core–shell structure during prolonged OER. Unlike most PBA-based catalysts with a single type of anion (e.g., S or P), this design establishes a dual-site M–S–P mechanism in which sulfur donates electrons to stabilize OH*/OOH* adsorption. At the same time, phosphorus withdraws electrons to prevent O* overbinding and accelerate O_2_ release, resulting in a balanced binding landscape that represents a conceptual advance. This design approach can be applied to other redox systems where synergistic interactions are essential to maximizing catalyst performance. The key takeaway from this study is that structural engineering goes far beyond aesthetics; it influences active sites, binding energy, electron transport, and durability—factors that ultimately dictate catalytic efficiency performance.

## Supplementary Material



## Notes

Detailed information on material synthesis, characterization, electrochemical measurements, FE-SEM, and STEM images, TGA, EDX, ICP, XPS, double-layer capacitance, Randles equivalent circuit, stability, and a comparison table (PDF)
