# Supplementary material for: Layered Porous Nanocubes: Harnessing Trimetallic PBA@WS2–Phosphorus Hybrid Architecture for Efficient Oxygen Evolution
Source: ACS Appl Mater Interfaces. 2026 Feb 18;18(10):15283–96. doi: 10.1021/acsami.6c01187 (PMC13006947; doi:10.1021/acsami.6c01187)
Supplement: Supplementary file 1 [file am6c01187_si_001.pdf]

Supporting Information

Layered Porous Nanocubes: Harnessing Trimetallic PBA@WS_2–Phosphorus Hybrid Architecture for Efficient Oxygen Evolution

Poulami Mukherjee,[a,b,] Krishnamoorthy Sathiyan,[a,c] Ronen Bar-Ziv,[d] Koichi Higashimine,[e] Toshiaki Taniike,[b] Arie Borenstein [a] and Tomer Zidki[a,*]*

[a] *Department of Chemical Sciences and the Centers for Radical Reactions and Materials Research, Ariel University, Ariel 407000, Israel.*

[b] *Graduate School of Advanced Science and Technology, Japan Advanced Institute of Science and Technology, 1-1 Asahidai, Nomi, Ishikawa, 923-1292, Japan.*

[c] *Institute of Advanced Energy, Kyoto University, Kyoto 611-0011, Japan.*

[d] *Department of Chemistry, Nuclear Research Centre, Negev, Beer-Sheva, Israel.*

[e] *The Center for Nano Materials and Technology, Japan Advanced Institute of Science and Technology, 1-1 Asahidai, Nomi, Ishikawa, 923-1292, Japan.*

*Corresponding authors

poulami@jaist.ac.jp

tomerzi@ariel.ac.il

Material characterization

The crystal structures were measured by X-ray diffraction (XRD) patterns recorded in the 2θ range of 5-80° (step size 0.02° and 5.0 s per step) using a Panalytical X'Pert Pro X-ray powder diffractometer with Cu Kα radiation (λ = 0.154 nm). The morphology was examined using field-emission scanning electron microscopy (FE-SEM, Tescan

MAIA3, accelerating voltage of 20 kV) and transmission electron microscopy (TEM, JEOL JEM-2100, accelerating voltage of 100 kV), both equipped with an energy-dispersive X-ray spectrometer (EDX). For SEM, the samples were affixed to carbon tape in a glove bag under a N_2 atmosphere. The TEM samples were dispersed in ethanol using ultrasonication for 15 minutes, then dropped onto a carbon-coated copper grid and left to dry naturally overnight. Thermogravimetric (TG) measurements were conducted in nitrogen at a temperature of 25-800 °C with a heating rate of 10 °C min^{-1} using a TA Q500 apparatus. The valence states of the surface elements were detected by X-ray photoelectron spectroscopy (XPS) using a Kratos AXIS Ultra DLD (Shimadzu, Kyoto, Japan) equipped with an Al–Kα anode. The samples were loaded into the holder with double-sided copper tape. Survey spectra were collected using a pass energy (P.E.) of 80 eV, and high-energy resolution spectra scans were collected with a pass energy of 160 eV. The acquired binding energies (BE) were taken with respect to the graphitic carbon C 1s peak at 284.6 eV. XPSPeak 4.1 software was used to deconvolute spectra and perform baseline correction using the Linear method. Post-reaction SEM images of the catalyst were obtained to examine morphological changes after catalysis. The GC loaded with the catalyst was collected after the reaction, and the catalyst was dispersed in ethanol by sonication. The resulting suspension was then drop-cast onto a silicon grid and allowed to dry overnight before SEM imaging.

Determination of the relative crystallinity of the synthesized samples using their XRD intensity data.

The crystallinity of each sample was estimated from its XRD intensity profile by integrating the area under the intensity vs. 2θ curve. This integral represents the total diffracted intensity, which correlates with the amount of crystalline material in the sample. For each sample, the area under the curve was calculated using the trapezoidal numerical integration method:

$$A = \int I(2\theta)\, d(2\theta) \quad \text{(S1)}$$

where:

- $I(2\theta)$is the recorded intensity,

- 2θ is the diffraction angle.

The sample with the largest total integrated area was taken as 100% crystallinity. The crystallinity of other samples was expressed relative to this value using:

$$Relative\ Intensity\ (\%) = \frac{A_{sample}}{A_{max}} \times 100 \quad (S2)$$

Electrochemical measurements

The electrochemical OER activity of the prepared catalysts was investigated in a 1.0 M KOH solution at pH ~14 (purged with N_2) using the PalmSens4 electrochemical workstation. During measurements, a conventional three-electrode setup was used, with a GCE as the working electrode, an Hg/HgO electrode as the reference electrode, and a graphite electrode as the counter electrode. To minimize capacitive current, the scan rate was set to 5 mVs^{-1} to acquire all linear-sweep voltammetry (LSV) curves without iR compensation. The potential reported in this work is referenced to the reversible hydrogen electrode (RHE) using the Nernst equation: E (RHE) = E (Hg/HgO) + 0.059 × pH + 0.098. The charging currents were measured from double-layer charging curves using cyclic voltammograms (CVs) at scan rates of 20-200 mVs^{-1} in the non-Faradaic potential range for OER, without redox processes. Electrochemical impedance spectroscopy (EIS) measurements were performed at an overpotential of 280 mV, spanning a frequency range from 100 kHz to 0.1 Hz (15 points per decade), with a sinusoidal perturbation amplitude of 10 mV. The best catalyst stability test was performed using 1,000 CV cycles at a scan rate of 50 mV s^{-1}, followed by chronoamperometry at current densities of 10 and 20 mA cm^{-2} for 24 hours, and 30 mA cm^{-2} for 15 hours. The Faradaic efficiency (FE) experiment was performed in a gas-tight H-cell, with a manometer connected to the anodic compartment. When a constant potential was applied during chronoamperometry, the change in the manometer's water level over time was observed. The recorded data is converted into moles of oxygen.

ECSA estimation

The electrochemical surface area (ECSA) of the catalysts was estimated from the double-layer capacitance (C_{dl}), derived from cyclic voltammograms (CVs) recorded in

a non-faradaic potential window between 1.02 and 1.12 V vs. RHE (**Figures S11a–d, S12a**). ECSA values were calculated using the relation:

$$ECSA = \frac{C_{dl}}{C_s} \text{ (S3)}$$

where C_s is the specific capacitance of a flat surface in 1.0 M KOH, taken as 40 μF cm^{-2} based on literature values for metal-based electrodes.[2,3] This approximation allows comparison of the relative active surface areas among the different catalyst architectures.

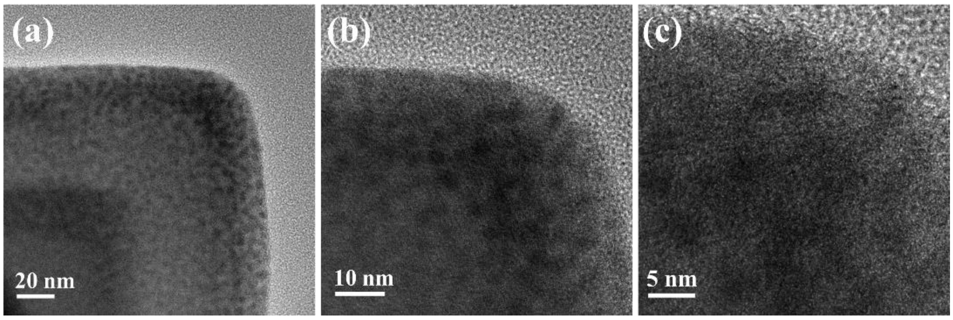

Figure S1. HR-TEM images of the Co-Co@Ni-Fe PBA showing the corner region of the nanoparticle at increasing magnification: (a) 20 nm scale, (b) 10 nm scale, and (c) 5 nm scale.

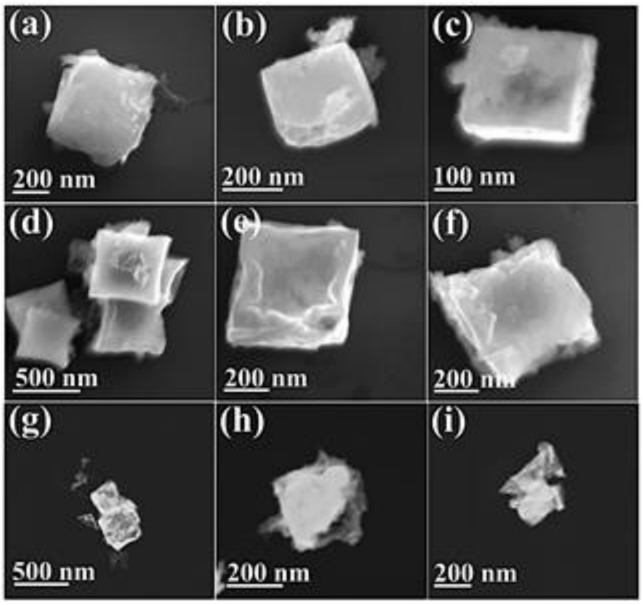

Figure S2. FE-SEM images of Co-Co@Ni-Fe PBA@WS_2-P phosphidated porous nanocubes (a-c) 2 h, (d-f) 4 h, (g-i) 6 h phosphidation durations.

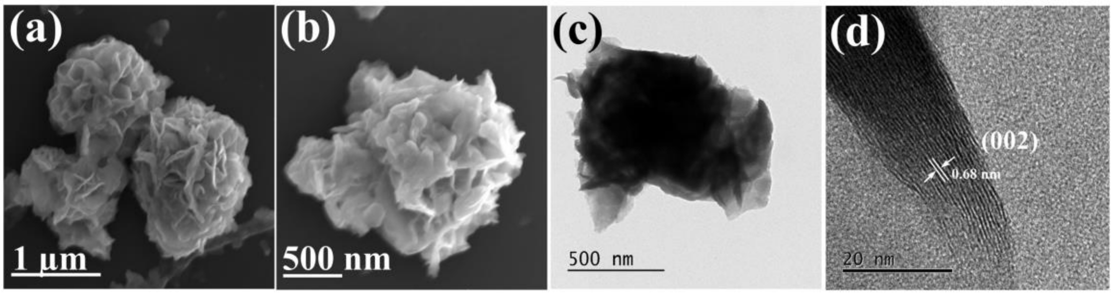

Figure S3. (a-b) FE-SEM images (c) Low magnification and (d) High-resolution TEM images of WS_2 nanoflowers

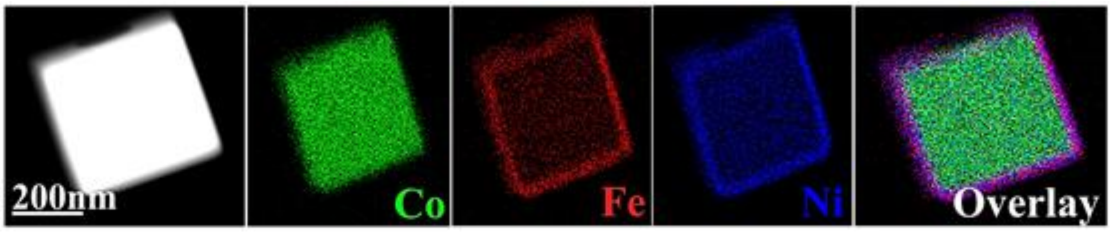

Figure S4. HAADF-STEM image of Co-Co@Ni-Fe core-shell PBA (nanoframe) and the corresponding elemental mapping of the same frame showing Co, Fe, and Ni elements with their overlay. The HAADF-STEM image and the corresponding elemental mapping are from our previous publication.[1]

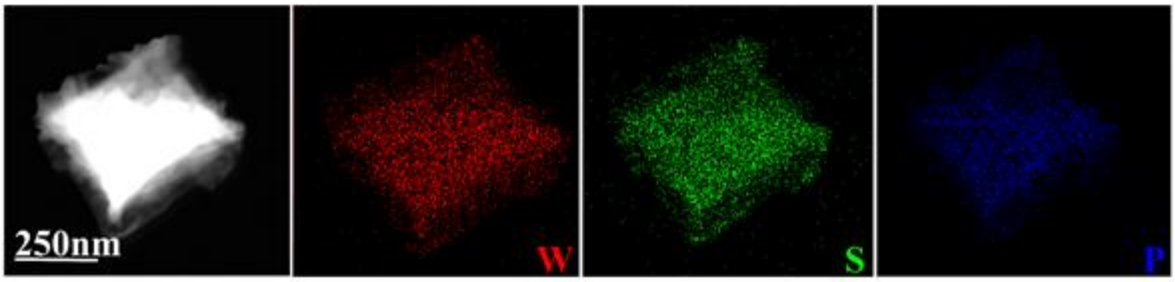

Figure S5. HAADF-STEM image of Co-Co@Ni-Fe PBA@WS_2-P phosphidated porous nanocubes and the corresponding elemental mapping of the same frame showing the uniform distribution of W, S, and P elements.

Table S1. Elemental mappings of Fe, Co, Ni, W, and S as determined by EDX analysis.

	Composition						
**Sample**	**Co (at. %)**	**Fe (at. %)**	**Ni (at. %)**	**W (at. %)**	**S+O (at. %)**	**P (at. %)**	**Ni/Fe**
Co-Co@Ni-Fe PBA@WS_2-P	30.7	5.3	6.4	2.8	50.5	4.4	1.2

Co-Co@Ni-Fe PBA@WS_2	32.1	5.5	6.7	2.9	52.8	-	1.2
Co-Co@Ni-Fe PBA	80.5	9.0	10.6	-	-	-	1.2

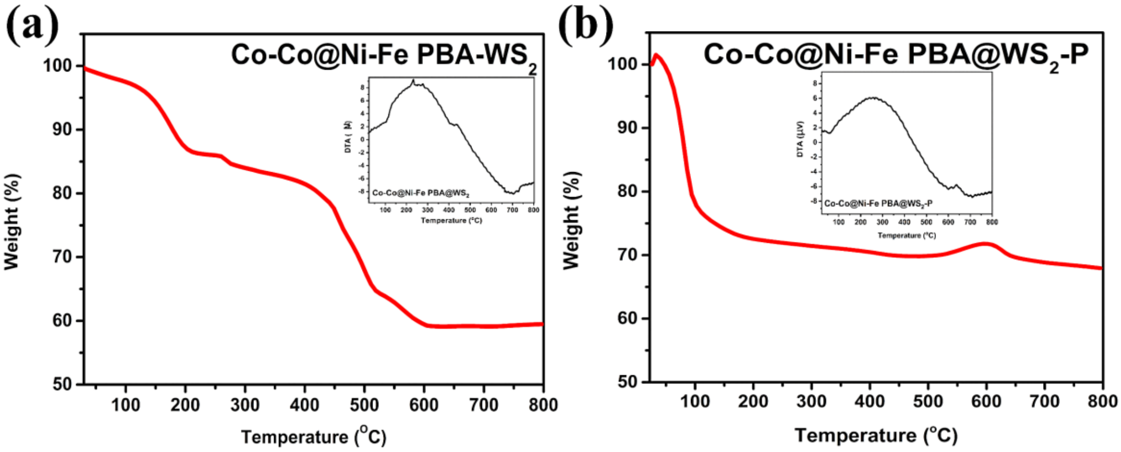

Figure S6. TGA and DTA inset of the (a) Co-Co@Ni-Fe PBA@WS_2 composites and (b) Co-Co@Ni-Fe PBA@WS_2-P phosphidated porous nanocubes in air.

Table S2. Summary of ICP-OES analysis

	Content (at. %)					
**Sample**	Co	Fe	Ni	W	P	Ni/Fe
Co-Co@Ni-Fe PBA@WS_2-P	30.34	21.89	33.58	7.52	6.67	1.53
Co-Co@Ni-Fe PBA@WS_2	34.65	23.12	34.40	7.83	-	1.48
Co-Co@Ni-Fe PBA	39.77	23.86	36.36	-	-	1.52
Co-Co PBA	100	-	-	-	-	-

Note: Elemental compositions obtained from EDX and ICP analyses are normalized to 100% based on the elements selected for quantification.

Table S3. Calculation of relative crystallinity based on integrated XRD intensity, normalized to the highest-intensity sample.

**Sample**	**Integrated Area (Intensity × 2θ°)**	**Relative Crystallinity (%)**
WS_2	13,407.26	12.69
Co–CoPBA	98,644.17	93.34
Co–Co@Ni–Fe PBA	88,939.77	84.16
Co–Co@Ni–Fe PBA@WS_2	105,678.83	100.00
Co–Co@Ni–Fe PBA@WS_2–P	90,344.27	85.49

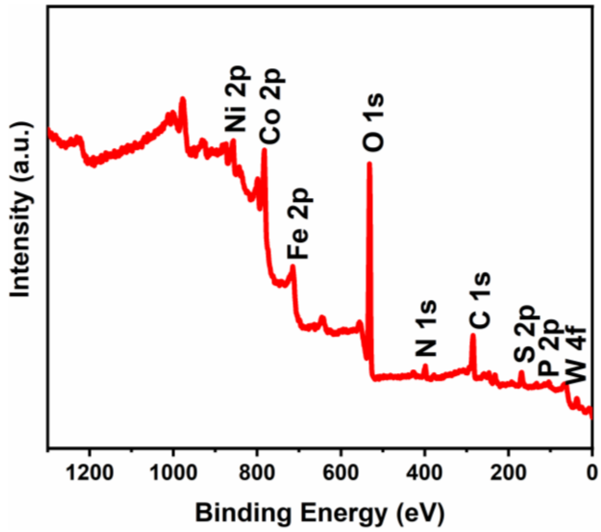

Figure S7. XPS survey spectrum of Co-Co@Ni-Fe PBA@WS_2-P phosphidated porous nanocubes.

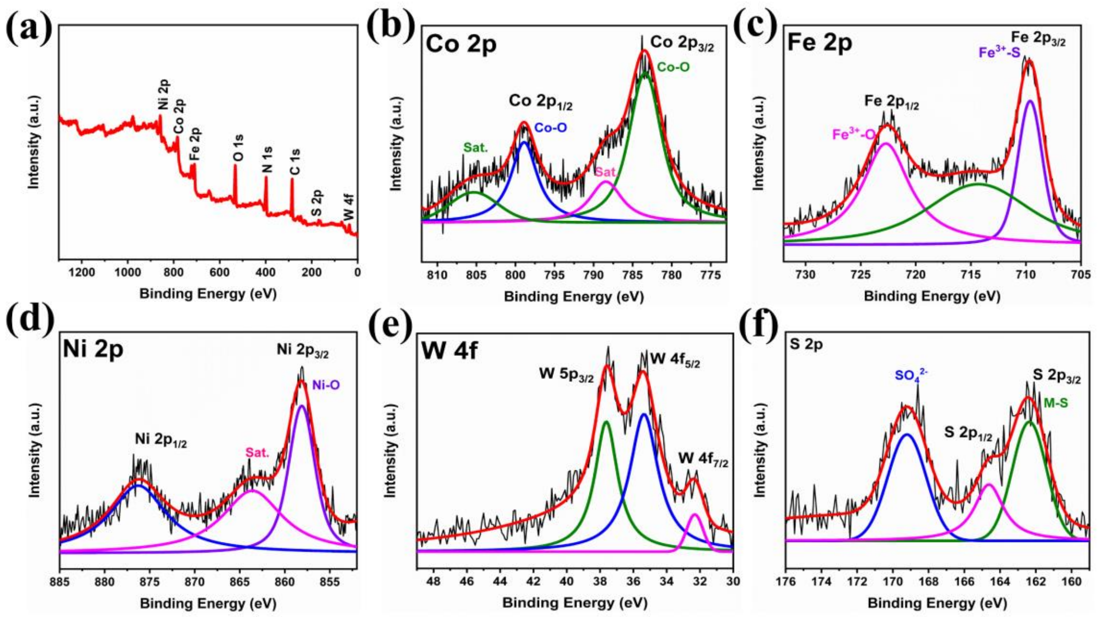

Figure S8. (a) XPS survey spectrum of Co-Co@Ni-Fe PBA@WS_2 composites, and high-resolution XPS spectra of (b) Co 2p, (c) Fe 2p, (d) Ni 2p, (e) W 2p, and (f) S 2p.

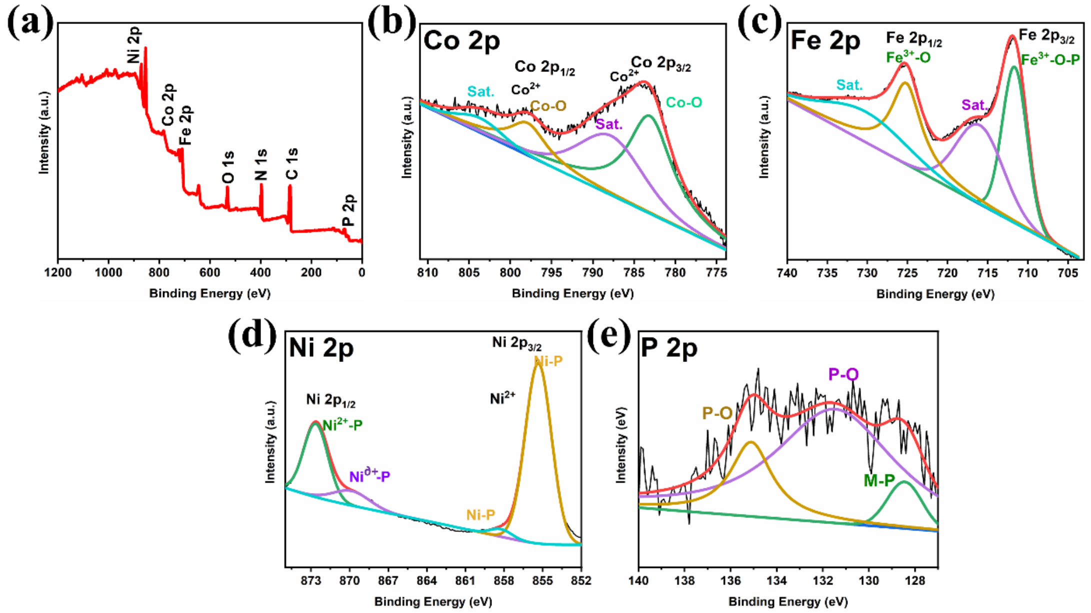

Figure S9. (a) XPS survey spectrum of Co-Co@Ni-Fe PBA-P, and high-resolution XPS spectra of (b) Co 2p, (c) Fe 2p, (d) Ni 2p, and (e) P 2p.

The survey spectrum of Co–Co@Ni–Fe PBA-P confirmed the presence of Fe, Co, Ni, O, C, N, and P (**Figure S9a**). In the Co 2p high-resolution XPS spectrum of the Co–Co@Ni–Fe PBA-P, the BE peaks centered at 783.3 and 798.3 eV were assigned to the spin orbitals of Co $2p_{3/2}$ and Co $2p_{1/2}$ in Co-O, which can be at both Co^{2+} or Co^{3+} oxidation states, **Figure S9b**. The satellite peaks at ~788.4 eV and ~804.2 eV are characteristic of Co^{2+} and Co^{3+} in the high-spin state. The peak positions are similar to

both Co–Co@Ni–Fe PBA@WS_2-P and Co–Co@Ni–Fe PBA@WS_2, indicating that the core electronic structure of cobalt remains broadly stable. In the Fe 2p spectrum (**Figure S9c**), the peaks at 725.2 eV and 711.72 eV can be assigned to the Fe $2p_{1/2}$ and Fe $2p_{3/2}$ orbitals of a highly oxidized iron species, Fe^{3+},[4] corresponding to Fe–P–O_x.[5] The BE peaks at 716.3 eV and 731.2 eV correspond to the typical Fe^{3+} shake-up signal.[4] For the Ni 2p spectrum, all four peaks at 872.6, 870.0, 858.3, and 855.3 eV suggest Ni-P bonds, **Figure S9d**.[6] The P 2p spectrum shows three dominant peaks, of which the peak positioned at 128.5 eV belongs to M-P.[5] In comparison, the other two peaks at 131.4 eV and 135.1 eV belong to oxidized phosphorus species due to surface exposure to air after phosphidation, **Figure S9e**.

Table S4. Summary of XPS quantitative analysis.

**Sample**	**Co (at. %)**	**Fe (at. %)**	**Ni (at. %)**	**W (at. %)**	**S (at. %)**	**P (at. %)**	**O (at. %)**
Co-Co@Ni-Fe PBA@WS_2-P	5.8	3.0	3.8	1.1	9.8	3.8	72.7
Co-Co@Ni-Fe PBA@WS_2	7.7	5.4	7.1	2.2	18.7	-	58.9
Co-Co@Ni-Fe PBA-P	6.9	16.2	30.6	-	-	4.2	42.1

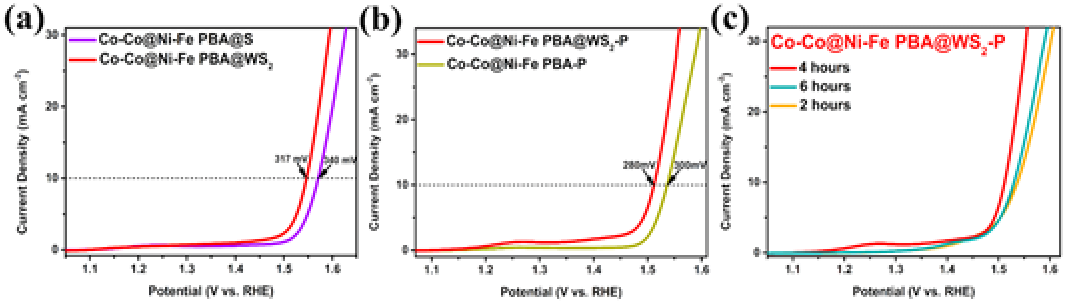

Figure S10. Comparison of LSV curves: (a) Co-Co@Ni-Fe PBA@S and Co-Co@Ni-Fe PBA@WS_2; (b) Co-Co@Ni-Fe PBA@WS_2-P and Co-Co@Ni-Fe PBA-P; (c) LSV curves of Co-Co@Ni-Fe PBA@WS_2-P recorded at different phosphidation durations.

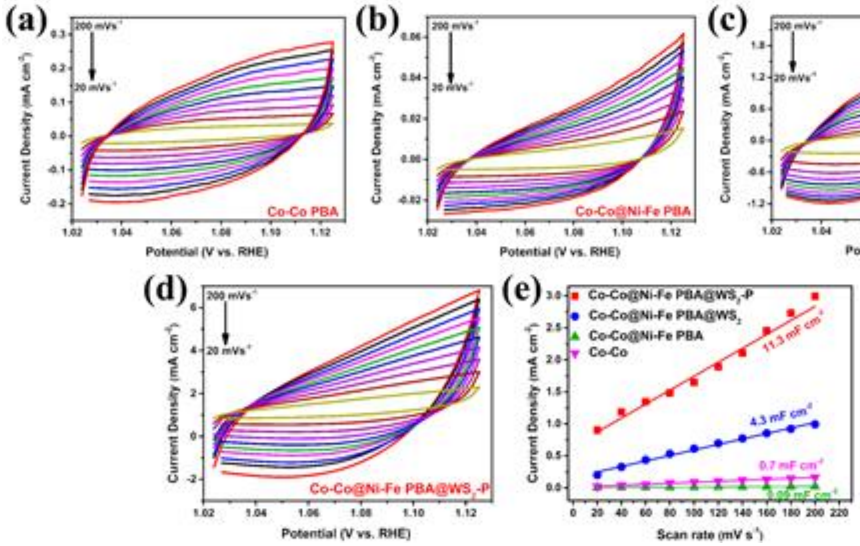

Figure S11. (a-d) Cyclic voltammograms of all the catalysts recorded at different scan rates from 200 to 20 mVs^{-1} in a 1.0 M KOH solution. (e) Capacitive current obtained from the cyclic voltammograms at 1.07 V vs. RHE as a function of scan rates.

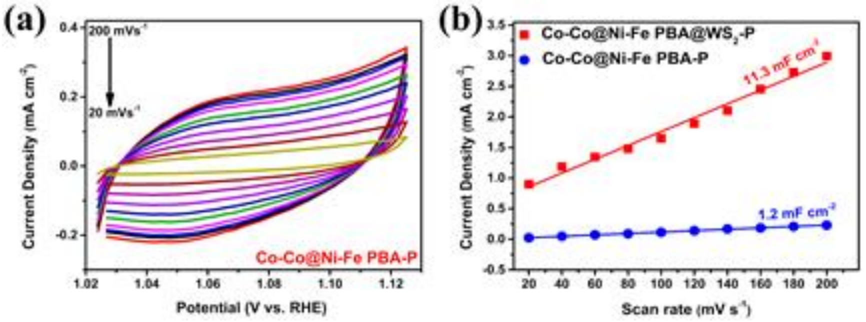

Figure S12. (a) Cyclic voltammograms of Co-Co@Ni-Fe PBA-P recorded at different scan rates from 200 to 20 mVs^{-1} in 1.0 M KOH solution. (b) Capacitive current obtained from the cyclic voltammograms at 1.07 V vs. RHE as a function of scan rates.

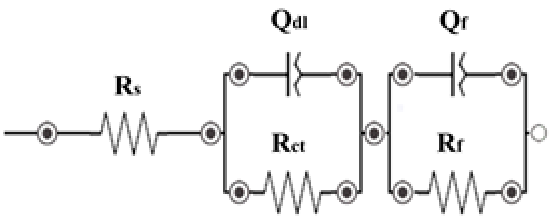

Figure S13. The Randles equivalent circuit model.

R_s denotes the uncompensated solution resistance, and Qdl is the constant phase element (CPE) associated with the electrochemical double-layer capacitance at the catalyst | electrolyte interface. R_{ct} represents the charge-transfer resistance at the interfaces. Q_f is the CPE associated with the capacitance of the catalyst thin film, and R_f is the total resistance at the electrode | catalyst interface and charge transfer within the catalyst film.[2]

Table S5. The fitting results of the impedance electrochemical element parameters of various samples.

Electrocatalysts	R_s	R_{ct}	Q_{dl}	R_f	Q_f
Co-Co@Ni-Fe PBA@WS_2-P	9.63	46.38	0.7521	9.64	0.7412
Co-Co@Ni-Fe PBA@WS_2	9.31	140.30	0.7419	9.31	0.7249
Co-Co@Ni-Fe PBA	7.14	2108.00	0.7832	7.15	0.7684
Co-Co PBA	9.05	1312.00	0.6814	10.22	0.6582

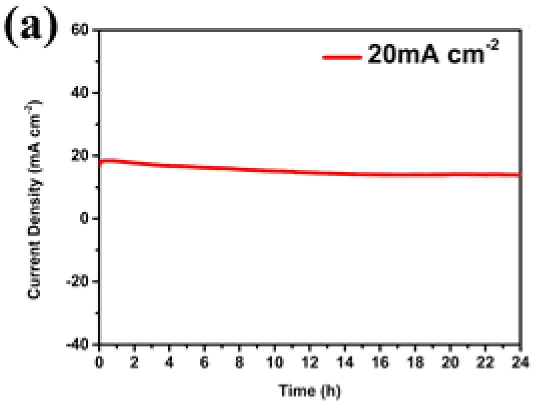

Figure S14. Chronopotentiometry response at the current density of 20 mA cm^{-2} for Co-Co@Ni-Fe PBA@WS_2-P phosphidated porous nanocubes.

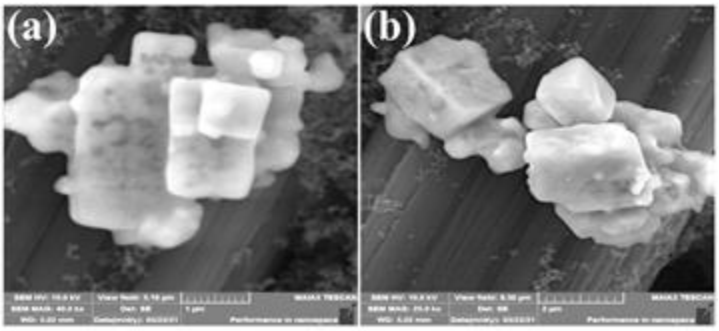

Figure S15. (a-b) FE-SEM images of Co-Co@Ni-Fe PBA@WS_2-P phosphidated porous nanocubes after 1,000 cycles.

Table S6. Elemental mappings of Co, Ni, Fe, W, S, P, and O as determined by EDX analysis before and after stability.

**Co-Co@Ni-Fe@WS_2-P**	**Before Stability**	**After Stability**
Elements	***Atomic %***	***Atomic %***
Co	27.9	24.2
Ni	6.1	5.4
Fe	4.0	3.5
W	3.9	3.5
S	6.2	5.5
P	22.2	20.4
O	29.7	37.5

Table S7. Comparison of catalytic parameters of different oxides, sulfides, and phosphides-based OER catalysts.

Catalysts	Electrolyte KOH (M)	Current density (mA cm^{-2})	Overpotential η_{10} (mV)	Tafel slope ($mV.dec^{-1}$)	Ref.
$Fe_{0.5}Ni_{0.5}Co_2O_4$	1	10	350	27	7
FeCo oxide	1	10	310	55.6	8
$Ni_xCo_{3-x}O_4$/NF	1	10	287	88	9
Ni-Co mixed oxide	1	10	380	50	10
Co_9S_8@MoS_2/CNFs	1	10	430	61	11
Co_9S_8/CNFs	1	10	512	78	11
$TiFe_2Co_2Ni_3MoS_{10}$	1	10	303	53.9	12
Co–FeS_2/CoS_2	1	10	278	73	13
Ni_3S_2/NF	1	10	296	65	14
M-MoS_2@Co	1	10	370	90	15
$Ni_{0.6}Co_{1.4}P$	1	10	300	80	16
NiFeP Cage@MS	1	10	290	46	17
Fe-NiCoP/PBA HNCs	1	10	290	70	18
NiCo/NiCoP	1	10	290	55	19
CoP-NPC	1	10	308	58.1	20
Oxygen-incorporated Ni_2P	0.1	10	347	63	21
$Co_{0.6}Fe_{0.4}P_{1.125}$	1	10	298	48	22
P-Co-Ni-S/NF	1	10	292	61.1	23
Sea-urchin structured Co-Fe-P	1	10	370	-	24
Co-doped nickel phosphides	1	10	360	65.7	25
NiCoP/C	1	10	330	96	26
CoP	1	10	320	71	27
Ni-P film	1	10	344	49	28

$Co_3(PO_4)_2$@N-C	1	10	317	62	29
$Ni_{0.6}Co_{1.4}(OH)_2$	1	10	300	80	16
NiS porous hollow microspheres	1	10	320	59	30
$(Ni_{0.62}Fe_{0.38})_2P$	1	10	290	44	31
PdNiP-H	1	10	300	48	32
CoP hollow polyhedron	1	10	400	57	33
FeP@CNT	1	10	300	53	34
$Fe_{1.1}Mn_{0.9}P$ nanorod	1	10	440	39	35
$Co_{0.68}Fe_{0.3}P$ polyhedrons	1	10	289	66	36
Needle-shaped Co_2P	1	10	310	50	37
Mn-Co oxyphosphide particles	1	10	320	52	38
Co_3FeP_xo	1	10	291	85	39
Ni_2P/NiO_x nanoparticles	1	10	290	59	40
Ce-$NiSe_2$/CoP	1	10	287	87.1	41
Ni-CoP	1	10	343	84	42
CoP@Co_3O_4/N-doped graphene	1	10	320	78.9	43
**Co-Co@Ni-Fe PBA@WS_2-P**	**1**	**10**	**280**	**70**	**This work**

References

(1) Mukherjee, P.; R. S., V.; Borenstein, A.; Zidki, T. Compositing Redox-Rich Co–Co@Ni–Fe PBA Nanocubes into Cauliflower-like Conducting Polypyrrole as an Electrode Material in Supercapacitors. *Mater Chem Front* 2023, *7* (6), 1110–1119. https://doi.org/10.1039/D2QM01162J.

(2) Mukherjee, P.; Sathiyan, K.; Vishwanath, R. S.; Zidki, T. Anchoring MoS_2 on an Ethanol-Etched Prussian Blue Analog for Enhanced Electrocatalytic Efficiency for the Oxygen Evolution Reaction. *Mater Chem Front* 2022, *6* (13), 1770–1778. https://doi.org/10.1039/D2QM00183G.

(3) Mukherjee, P.; Sathiyan, K.; Bar-Ziv, R.; Zidki, T. Chemically Etched Prussian Blue Analog–WS_2 Composite as a Precatalyst for Enhanced Electrocatalytic Water Oxidation in Alkaline Media. *Inorg Chem* 2023, *62* (35), 14484–14493. https://doi.org/10.1021/acs.inorgchem.3c02537.

(4) González-Banciella, A.; Martinez-Diaz, D.; Kundu, M.; Sánchez, M.; Ureña, A. Cu-Doped MOF-Derived α-Fe_2O_3 Coatings on Carbon Fiber Fabric as Li-Ion and Na-Ion Battery Anodes for Potential Structural Batteries. *J Power Sources* 2025, *630*, 236071. https://doi.org/10.1016/j.jpowsour.2024.236071.

(5) Yun, W. H.; Das, G.; Kim, B.; Park, B. J.; Yoon, H. H.; Yoon, Y. S. Ni–Fe Phosphide Deposited Carbon Felt as Free-Standing Bifunctional Catalyst Electrode for Urea Electrolysis. *Sci Rep* 2021, *11* (1), 22003. https://doi.org/10.1038/s41598-021-01383-3.

(6) Wang, Z.; Li, L.; Liu, M.; Miao, T.; Ye, X.; Meng, S.; Chen, S.; Fu, X. A New Phosphidation Route for the Synthesis of NiP and Their Cocatalytic Performances for Photocatalytic Hydrogen Evolution over G-C_3N_4. *Journal of Energy Chemistry* 2020, *48*, 241–249. https://doi.org/10.1016/j.jechem.2020.01.017.

(7) Yan, K.-L.; Shang, X.; Li, Z.; Dong, B.; Li, X.; Gao, W.-K.; Chi, J.-Q.; Chai, Y.-M.; Liu, C.-G. Ternary Mixed Metal Fe-Doped $NiCo_2O_4$ Nanowires as Efficient Electrocatalysts for Oxygen Evolution Reaction. *Appl Surf Sci* 2017, *416*, 371–378. https://doi.org/10.1016/j.apsusc.2017.04.204.

(8) Chuang, C.-H.; Hsiao, L.-Y.; Yeh, M.-H.; Wang, Y.-C.; Chang, S.-C.; Tsai, L.-D.; Ho, K.-C. Prussian Blue Analogue-Derived Metal Oxides as Electrocatalysts for Oxygen Evolution Reaction: Tailoring the Molar Ratio of Cobalt to Iron. *ACS Appl Energy Mater* 2020, *3* (12), 11752–11762. https://doi.org/10.1021/acsaem.0c01903.

(9) Shen, Y.; Guo, S.-G.; Du, F.; Yuan, X.-B.; Zhang, Y.; Hu, J.; Shen, Q.; Luo, W.; Alsaedi, A.; Hayat, T.; Wen, G.; Li, G.-L.; Zhou, Y.; Zou, Z. Prussian Blue Analogue-Derived Ni and Co Bimetallic Oxide Nanoplate Arrays Block-Built from Porous and Hollow Nanocubes for the Efficient Oxygen Evolution Reaction. *Nanoscale* 2019, *11* (24), 11765–11773. https://doi.org/10.1039/C9NR01804B.

(10) Han, L.; Yu, X.-Y. Y.; Lou, X. W. D. (David). Formation of Prussian-Blue-Analog Nanocages via a Direct Etching Method and Their Conversion into Ni-Co-Mixed Oxide for Enhanced Oxygen Evolution. *Advanced Materials* 2016, *28* (23), 4601–4605. https://doi.org/10.1002/adma.201506315.

(11) Zhu, H.; Zhang, J.; Yanzhang, R.; Du, M.; Wang, Q.; Gao, G.; Wu, J.; Wu, G.; Zhang, M.; Liu, B.; Yao, J.; Zhang, X. When Cubic Cobalt Sulfide Meets Layered Molybdenum Disulfide: A Core–Shell System Toward Synergetic Electrocatalytic Water Splitting. *Advanced Materials* 2015, *27* (32), 4752–4759. https://doi.org/10.1002/adma.201501969.

(12) Lin, L.; Ding, Z.; Karkera, G.; Diemant, T.; Kante, M. V.; Agrawal, D.; Hahn, H.; Aghassi-Hagmann, J.; Fichtner, M.; Breitung, B.; Schweidler, S. High-Entropy Sulfides as Highly Effective Catalysts for the Oxygen Evolution Reaction. *Small Struct* 2023, *4* (9). https://doi.org/10.1002/sstr.202300012.

(13) Wang, K.; Guo, W.; Yan, S.; Song, H.; Shi, Y. Hierarchical Co–FeS_2/CoS_2 Heterostructures as a Superior Bifunctional Electrocatalyst. *RSC Adv* 2018, *8* (50), 28684–28691. https://doi.org/10.1039/C8RA05237A.

(14) Li, L.; Sun, C.; Shang, B.; Li, Q.; Lei, J.; Li, N.; Pan, F. Tailoring the Facets of Ni_3S_2 as a Bifunctional Electrocatalyst for High-Performance Overall Water-Splitting. *J Mater Chem A Mater* 2019, *7* (30), 18003–18011. https://doi.org/10.1039/C9TA05578A.

(15) Cao, S.; Wu, W.; Liu, C.; Song, L.; Xu, Q.; Zhang, H.; Zhao, Y. Supramolecular Macrocycle Regulated Single-Atom MoS_2@Co Catalysts for Enhanced Oxygen Evolution Reaction. *Energy & Environmental Materials* 2024, *7* (5). https://doi.org/10.1002/eem2.12702.

(16) Qiu, B.; Cai, L.; Wang, Y.; Lin, Z.; Zuo, Y.; Wang, M.; Chai, Y. Fabrication of Nickel-Cobalt Bimetal Phosphide Nanocages for Enhanced Oxygen Evolution Catalysis. *Adv Funct Mater* 2018, *28* (17), 1706008. https://doi.org/10.1002/adfm.201706008.

(17) Li, J.; Zheng, J.; Cheng, X.; Yue, G.; Luo, X. NiFeP Nanocages Embedded in Melamine Sponge Derived Nitrogen Doped Porous Carbon Foam as an Efficient Oxygen Evolution Electrocatalyst. *J Solid State Chem* 2019, *278*, 120881. https://doi.org/10.1016/j.jssc.2019.07.042.

(18) Li, D.; Liu, C.; Ma, W.; Xu, S.; Lu, Y.; Wei, W.; Zhu, J.; Jiang, D. Fe-Doped NiCoP/Prussian Blue Analog Hollow Nanocubes as an Efficient Electrocatalyst for Oxygen Evolution Reaction. *Electrochim Acta* 2020, 137492. https://doi.org/10.1016/j.electacta.2020.137492.

(19) Shao, Z.; Qi, H.; Wang, X.; Sun, J.; Guo, N.; Huang, K.; Wang, Q. Boosting Oxygen Evolution by Surface Nitrogen Doping and Oxygen Vacancies in Hierarchical NiCo/NiCoP Hybrid Nanocomposite. *Electrochim Acta* 2019, *296*, 259–267. https://doi.org/10.1016/j.electacta.2018.11.006.

(20) Zhou, X.; Xu, L.; Gao, Y.; Li, L.; Tang, J.; Yang, J. Phosphorization of a Prussian Blue Analogue-Derived Co–N–C Catalyst for Synchronously Boosting the Oxygen Reduction and Evolution Reactions. *Sustain Energy Fuels* 2020, *4* (5), 2411–2421. https://doi.org/10.1039/D0SE00156B.

(21) Li, Z.; Dou, X.; Zhao, Y.; Wu, C. Enhanced Oxygen Evolution Reaction of Metallic Nickel Phosphide Nanosheets by Surface Modification. *Inorg Chem Front* 2016, *3* (8), 1021–1027. https://doi.org/10.1039/C6QI00078A.

(22) Lian, Y.; Sun, H.; Wang, X.; Qi, P.; Mu, Q.; Chen, Y.; Ye, J.; Zhao, X.; Deng, Z.; Peng, Y. Carved Nanoframes of Cobalt–Iron Bimetal Phosphide as a

Bifunctional Electrocatalyst for Efficient Overall Water Splitting. *Chem Sci* 2019, *10* (2), 464–474. https://doi.org/10.1039/C8SC03877E.

(23) Zhang, F.; Ge, Y.; Chu, H.; Dong, P.; Baines, R.; Pei, Y.; Ye, M.; Shen, J. Dual-Functional Starfish-like P-Doped Co–Ni–S Nanosheets Supported on Nickel Foams with Enhanced Electrochemical Performance and Excellent Stability for Overall Water Splitting. *ACS Appl Mater Interfaces* 2018, *10* (8), 7087–7095. https://doi.org/10.1021/acsami.7b18403.

(24) Mendoza-Garcia, A.; Su, D.; Sun, S. Sea Urchin-like Cobalt–Iron Phosphide as an Active Catalyst for Oxygen Evolution Reaction. *Nanoscale* 2016, *8* (6), 3244–3247. https://doi.org/10.1039/C5NR08763E.

(25) Li, J.; Yan, M.; Zhou, X.; Huang, Z.-Q.; Xia, Z.; Chang, C.-R.; Ma, Y.; Qu, Y. Mechanistic Insights on Ternary $Ni_{2-x}Co_xP$ for Hydrogen Evolution and Their Hybrids with Graphene as Highly Efficient and Robust Catalysts for Overall Water Splitting. *Adv Funct Mater* 2016, *26* (37), 6785–6796. https://doi.org/10.1002/adfm.201601420.

(26) He, P.; Yu, X.-Y.; Lou, X. W. D. Carbon-Incorporated Nickel-Cobalt Mixed Metal Phosphide Nanoboxes with Enhanced Electrocatalytic Activity for Oxygen Evolution. *Angewandte Chemie International Edition* 2017, *56* (14), 3897–3900. https://doi.org/10.1002/anie.201612635.

(27) Chang, J.; Xiao, Y.; Xiao, M.; Ge, J.; Liu, C.; Xing, W. Surface Oxidized Cobalt-Phosphide Nanorods As an Advanced Oxygen Evolution Catalyst in Alkaline Solution. *ACS Catal* 2015, *5* (11), 6874–6878. https://doi.org/10.1021/acscatal.5b02076.

(28) Jiang, N.; You, B.; Sheng, M.; Sun, Y. Bifunctionality and Mechanism of Electrodeposited Nickel-Phosphorous Films for Efficient Overall Water Splitting. *ChemCatChem* 2016, *8* (1), 106–112. https://doi.org/10.1002/cctc.201501150.

(29) Yuan, C.-Z.; Jiang, Y.-F.; Wang, Z.; Xie, X.; Yang, Z.-K.; Yousaf, A. Bin; Xu, A.-W. Cobalt Phosphate Nanoparticles Decorated with Nitrogen-Doped Carbon Layers as Highly Active and Stable Electrocatalysts for the Oxygen Evolution Reaction. *J Mater Chem A Mater* 2016, *4* (21), 8155–8160. https://doi.org/10.1039/C6TA01929C.

(30) Luo, P.; Zhang, H.; Liu, L.; Zhang, Y.; Deng, J.; Xu, C.; Hu, N.; Wang, Y. Targeted Synthesis of Unique Nickel Sulfide (NiS, NiS_2) Microarchitectures and the Applications for the Enhanced Water Splitting System. *ACS Appl Mater Interfaces* 2017, *9* (3), 2500–2508. https://doi.org/10.1021/acsami.6b13984.

(31) Zou, H.-H.; Yuan, C.-Z.; Zou, H.-Y.; Cheang, T.-Y.; Zhao, S.-J.; Qazi, U. Y.; Zhong, S.-L.; Wang, L.; Xu, A.-W. Bimetallic Phosphide Hollow Nanocubes Derived from a Prussian-Blue-Analog Used as High-Performance Catalysts for the Oxygen Evolution Reaction. *Catal Sci Technol* 2017, *7* (7), 1549–1555. https://doi.org/10.1039/C7CY00035A.

(32) Sankar, S.; Sugawara, Y.; Assa Aravindh, S.; Jose, R.; Tamaki, T.; Anilkumar, G. M.; Yamaguchi, T. Tuning Palladium Nickel Phosphide toward Efficient Oxygen Evolution Performance. *ACS Appl Energy Mater* 2020, *3* (1), 879–888. https://doi.org/10.1021/acsaem.9b01996.

(33) Liu, M.; Li, J. Cobalt Phosphide Hollow Polyhedron as Efficient Bifunctional Electrocatalysts for the Evolution Reaction of Hydrogen and Oxygen. *ACS Appl Mater Interfaces* 2016, *8* (3), 2158–2165. https://doi.org/10.1021/acsami.5b10727.

(34) Yan, Y.; Zhao, B.; Yi, S. C.; Wang, X. Assembling Pore-Rich FeP Nanorods on the CNT Backbone as an Advanced Electrocatalyst for Oxygen Evolution. *J Mater Chem A Mater* 2016, *4* (33), 13005–13010. https://doi.org/10.1039/C6TA05317C.

(35) Li, D.; Baydoun, H.; Kulikowski, B.; Brock, S. L. Boosting the Catalytic Performance of Iron Phosphide Nanorods for the Oxygen Evolution Reaction by Incorporation of Manganese. *Chemistry of Materials* 2017, *29* (7), 3048–3054. https://doi.org/10.1021/acs.chemmater.7b00055.

(36) Li, F.; Bu, Y.; Lv, Z.; Mahmood, J.; Han, G.-F.; Ahmad, I.; Kim, G.; Zhong, Q.; Baek, J.-B. Porous Cobalt Phosphide Polyhedrons with Iron Doping as an Efficient Bifunctional Electrocatalyst. *Small* 2017, *13* (40), 1701167. https://doi.org/10.1002/smll.201701167.

(37) Dutta, A.; Samantara, A. K.; Dutta, S. K.; Jena, B. K.; Pradhan, N. Surface-Oxidized Dicobalt Phosphide Nanoneedles as a Nonprecious, Durable, and Efficient OER Catalyst. *ACS Energy Lett* 2016, *1* (1), 169–174. https://doi.org/10.1021/acsenergylett.6b00144.

(38) Guan, B. Y.; Yu, L.; Lou, X. W. D. General Synthesis of Multishell Mixed-Metal Oxyphosphide Particles with Enhanced Electrocatalytic Activity in the Oxygen Evolution Reaction. *Angewandte Chemie International Edition* 2017, *56* (9), 2386–2389. https://doi.org/10.1002/anie.201611804.

(39) Babu, D. D.; Huang, Y.; Anandhababu, G.; Ghausi, M. A.; Wang, Y. Mixed-Metal–Organic Framework Self-Template Synthesis of Porous Hybrid Oxyphosphides for Efficient Oxygen Evolution Reaction. *ACS Appl Mater Interfaces* 2017, *9* (44), 38621–38628. https://doi.org/10.1021/acsami.7b13359.

(40) Stern, L.-A.; Feng, L.; Song, F.; Hu, X. Ni_2P as a Janus Catalyst for Water Splitting: The Oxygen Evolution Activity of Ni_2P Nanoparticles. *Energy Environ Sci* 2015, *8* (8), 2347–2351. https://doi.org/10.1039/C5EE01155H.

(41) Jiang, W.; Zhao, B.; Li, Z.; Zhou, P.; Zhao, Y.; Chen, X.; Wang, J.; Yang, R.; Zuo, C. Multihole Ce-Doped $NiSe_2$/CoP Hybrid Nanosheets for Improved Electrocatalytic Alkaline Water and Simulative Seawater Oxidation. *Int J Hydrogen Energy* 2024, *73*, 590–597. https://doi.org/10.1016/j.ijhydene.2024.06.111.

(42) Park, D.-H.; Kim, M.-H.; Kim, M.; Byeon, J.-H.; Jang, J.-S.; Kim, J.-H.; Lim, D.-M.; Park, S.-H.; Gu, Y.-H.; Kim, J.; Park, K.-W. Spherical Nickel Doped Cobalt Phosphide as an Anode Catalyst for Oxygen Evolution Reaction in Alkaline Media: From Catalysis to System. *Appl Catal B* 2023, *327*, 122444. https://doi.org/10.1016/j.apcatb.2023.122444.

(43) Meng, X.-C.; Luan, J.; Liu, Y.; Sheng, Y.-S.; Guo, F.-Y.; Zheng, P.; Duan, W.-L.; Li, W.-Z. Co-MOF-Derived Core–Shell CoP@Co_3O_4 Nanoparticle Loaded N-Doped Graphene: An Efficient Catalyst for the Oxygen Evolution Reaction. *J Mater Chem A Mater* 2025, *13* (1), 627–637. https://doi.org/10.1039/D4TA07696F.
